# The fluorescence‐activating and absorption‐shifting tag (FAST), a versatile protein marker for live plant cell imaging

**DOI:** 10.1111/tpj.70966

**Published:** 2026-06-12

**Authors:** Pascale David, Morgane Michaud, Lucas Moyet, Sara Pullara, Benoit Lacombe, Jinsheng Zhu, Arnaud Gautier, Thierry Desnos, Edouard Bertrand, Norbert Rolland, Laurent Nussaume

**Affiliations:** ^1^ Aix Marseille Univ, CEA, CNRS, BIAM, UMR7265, EBMP Saint‐Paul Lez Durance 13115 France; ^2^ Laboratoire de Physiologie Cellulaire et Végétale Université Grenoble Alpes, INRAE, CNRS, CEA, IRIG, CEA‐Grenoble 17 rue des Martyrs Grenoble 38000 France; ^3^ IPSiM, Université de Montpellier, CNRS, INRAE, Institut Agro Montpellier Place Viala Montpellier Cedex 34060 France; ^4^ Sorbonne Université, École Normale Supérieure, Université PSL, CNRS, Laboratoire des Biomolécules, LBM Paris 75005 France; ^5^ Institute of Human Genetics, Montpellier Univ‐CNRS, UMR 9002 Montpellier Cedex 05 34094 France

**Keywords:** Arabidopsis, FAST, fluorescent marker, fluorogen, Split‐FAST

## Abstract

The development of real‐time imaging has progressed spectacularly in recent years, largely due to the availability of multiple fluorophores with distinct spectral properties. This progress enables the simultaneous imaging of an increasing number of targets, providing access to complex biological processes occurring during development, signaling, and metabolism. In this study, we successfully tested the transfer of three different fluorescence‐activating and absorption‐shifting tags (FAST), originally developed in animal systems and yeast, to plants. These small (14 kDa) proteins associate non‐covalently and reversibly with specific chemicals known as fluorogens, generating bright fluorescence signals that can be reversibly eliminated by washing. This property offers versatile applications. Green‐ and Red‐FAST proteins were successfully used to label proteins targeted to several plant cellular compartments. In contrast, the latest generation of FAST proteins, identified in *Rheinheimera* sp. A13L (*RspA*), which accepts multiple fluorogens and offers broad versatility in excitation and emission spectra, was used with a split FAST version to demonstrate cytosolic protein–protein interactions.

## Significance Statement

Benefits of fluorescence‐activating and absorption‐shifting tag (FAST) as a novel reversible multi‐color fluorescent reporter system to monitor protein location or protein–protein interactions.

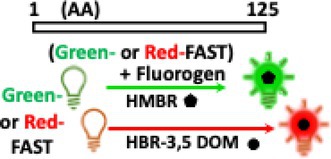

Fluorescent reporter proteins play a crucial role in plant cell biology. This study introduces fluorescence‐activating and absorption‐shifting tags (FAST), which enable reversible, multi‐color, real‐time imaging of proteins, expanding the available toolkit for monitoring protein localization and interactions and thereby deepening our understanding of complex cellular processes in plants.

## INTRODUCTION

Cell biology relies on precise spatiotemporal characterization of protein localization and regulation, two critical parameters for elucidating complex molecular and cellular processes. Reporter gene fusions constitute a central methodological approach for such investigations. One of the earliest markers employed in plant biology was the *Escherichia coli* gene encoding β‐glucuronidase (GUS), a highly stable enzyme that catalyzes the conversion of the colorless substrate X‐Gluc (5‐bromo‐4‐chloro‐3‐indolyl glucuronide) into an insoluble blue precipitate (Jefferson et al., 1987). Owing to its high sensitivity, this technique has been extensively used to study gene expression patterns and plant development (Dolan et al., 1993; Eastmond et al., 2000; Klimyuk et al., 1995; Reymond et al., 2006). However, its major limitation lies in the lethality of the staining procedure, which precludes live imaging.

This constraint was overcome with the discovery of the Green Fluorescent Protein (GFP) (Chalfie et al., 1994; Minsky, 1988; Prasher et al., 1992; Tsien, 1998). GFP enables non‐invasive fluorescent labeling in living multicellular organisms and, when combined with advanced microscopy techniques, has revolutionized live‐cell imaging. GFP and its numerous engineered variants provide a wide range of fluorescence properties, including distinct excitation and emission spectra and varying brightness levels, thereby allowing adaptation to specific experimental requirements (Ellenberg et al., 1999). Moreover, GFP can be used in combination with other fluorescent tags and markers, facilitating multiplex labeling strategies and co‐localization analyses (Bayle et al., 2011; Kanno et al., 2016; Parizot et al., 2008).

GFP has become firmly established across diverse experimental systems, ranging from cell culture to complex multicellular organisms and *in vitro* assays. Its widespread adoption has contributed to improved reproducibility and comparability of results across laboratories. Beyond protein localization, GFP fusion proteins enable the investigation of protein dynamics in physiologically relevant contexts, including turnover, polarization, intracellular trafficking, and protein–protein interactions (Chalfie et al., 1994; Tsien, 1998). Importantly, such applications require that the GFP fusion does not significantly interfere with the biological activity, function, or stability of the tagged protein. GFP‐based approaches have also been instrumental in visualizing complex cellular processes, such as *in vivo* transcriptional activity (Bertrand et al., 1998; Hani et al., 2021). These studies benefit from compatibility with a broad array of imaging modalities, including confocal, spinning‐disk, two‐photon, light‐sheet, and super‐resolution microscopy.

Overall, GFP tagging is a widely used and powerful tool in cell biology. Nevertheless, it presents several well‐documented limitations (for review, see Wiedenmann et al., 2009). GFP is a relatively large protein (~27 kDa), and fusion to a target protein substantially increases the overall molecular size. Combined with its tendency to oligomerize, this size increase may alter the biochemical properties, localization, or functionality of the tagged protein (Wiedenmann et al., 2009). In addition, GFP fluorescence strictly depends on molecular oxygen, which precludes its use under anaerobic conditions (Remington, 2006). The fluorescence of most GFP variants is also sensitive to pH fluctuations (Shaner et al., 2005), limiting their applicability for studying acidic cellular compartments, such as the plant vacuole. Furthermore, the relatively long maturation time of many GFP variants, up to 1 h, hinders the observation of rapid cellular processes. Fixation procedures are known to weaken or even abolish GFP fluorescence (Kusser & Randall, 2003). Temperature variations and protease activity may also generate artifacts, as cleaved GFP can accumulate in the nucleoplasm and cytoplasm (Haseloff et al., 1997). Finally, GFP and its derivatives are highly susceptible to photobleaching, whereby prolonged exposure to excitation light leads to irreversible fluorescence loss, thereby limiting long‐term live‐cell imaging experiments. Consequently, alternative fluorescent reporters are of considerable interest to overcome these limitations.

**Figure 1:**
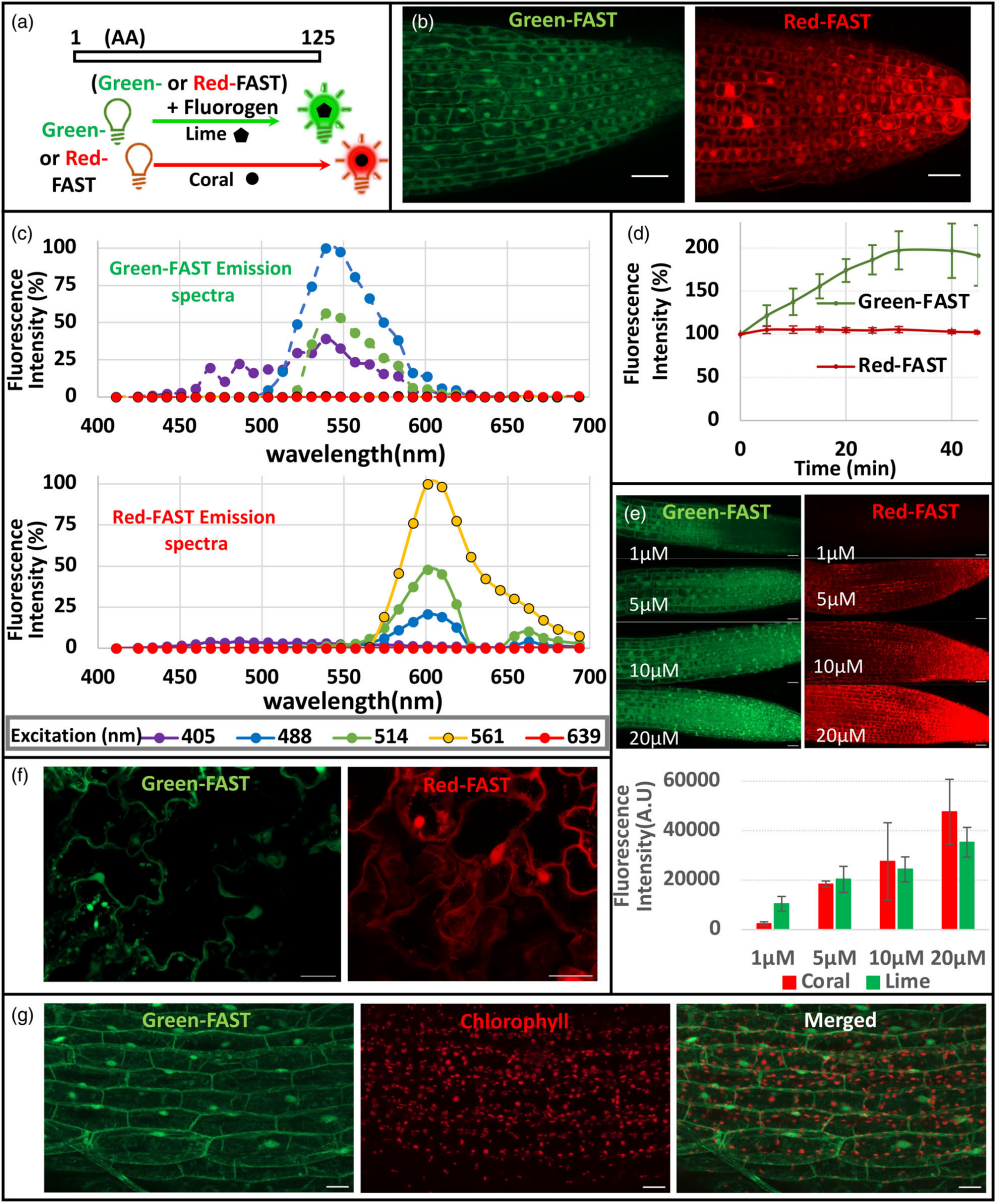
Analysis of the fluorescence signal emitted by Green‐ and Red‐FAST expressed in plants fused to constitutive 35S promoter with their respective Lime and Coral fluorogens. (a) Diagram illustrating how FAST works. (b) Fluorescence resulting from stable constitutive expression of Green‐ or Red‐FAST protein in the root in the presence of their respective fluorogens (15 μM) incubated 35 min normalized to the maximum intensity measured. (c) Emission spectra of Green‐FAST (Laser 1%; Detector gain 799 V) and Red‐FAST (Laser 0.8%; Detector Gain 850 V) in response to different excitations performed on the same root nuclei (the normalization is performed relative to the excitation peak giving the maximum signal), seedlings are incubated for 35 min with the fluorogen and imaged with LSM980 Airyscan2. (d) Evolution of the fluorescence intensity according to incubation time with the fluorogens in the roots (samples are normalized according to fluorescence intensity detected at *T*_0_ when the first observation was performed after 5 min incubation with the fluorogen (Lime 10 μM or Coral 15 μM)). Seedlings remaining in the fluorogen staining solution are then imaged every 5 min over a 45‐min period. Laser 488 nm 0.8% for Green‐FAST and laser 561 nm 0.4% for Red‐FAST. (e) Variation of the labeling intensity according to the concentrations of fluorogen used. Seedlings incubated 35 mins in their respective fluorogen at different concentrations. Laser 488 nm 0.2% Detector gain 750 V Emission spectra 491–632 nm for Green‐FAST and for Red‐FAST: Laser 561 nm 0.8% Detector gain 850 V Emission spectra 588–641 nm. (f) Expression of Green‐ or Red‐FAST proteins in leaves after an incubation of 35 min with the fluorogens at 15 μM. (g) Expression of Green‐FAST proteins in the hypocotyl (Laser 488 nm 0.2% Detector gain 750 V Emission spectra 491–632 nm) combined with chlorophyll fluorescence emission (laser 639 nm 2% Detector gain 700 V Emission spectra 654–713 nm) after an incubation of 35 min with the fluorogen at 10 μM. Scale bar: 25 μm. We used Zeiss Plan‐Apochromat 20X/0.8 M27 objective for (d, e, g) and LD LCI Plan‐Apochromat 40X/1.2 Imm Kor DIC M27 for (f). Three different ROI were imaged for each experiment (d–e), in triplicate. The error bars on the graphs represent the standard deviations.

In this context, the recent development of Fluorescence‐Activating and Absorption‐Shifting Tag (FAST) proteins represents a promising alternative to GFP‐based systems (Plamont et al., 2016). The FAST system (Figure 1a) consists of two components: a protein tag (FAST) binding a small organic fluorogenic molecule named fluorogen. FAST proteins were originally engineered from the Photoactive Yellow Protein (PYP) identified in *Halorhodospira halophila*. They bind rapidly and reversibly to a range of 4‐hydroxybenzylidene rhodamine–derived fluorogens (Plamont et al., 2016).

Notably, the FAST protein ligand is relatively small (~14 kDa), approximately half the size of GFP, thereby minimizing the steric impact of protein fusion. The fluorogen alone exhibits a very low fluorescence quantum yield and therefore emits minimal signal upon excitation. However, binding to the FAST protein induces a strong fluorescence enhancement by stabilizing the fluorogen within a specific binding cavity. The affinity of the different fluorogen–protein pairs has been reported with dissociation constants (Kd) ranging from the micromolar to submicromolar range (Plamont et al., 2016; Tebo & Gautier, 2019). Similar to GFP, multiple FAST variants and corresponding fluorogens have been developed (https://www.the‐twinkle‐factory.com/), providing fluorescent markers with diverse excitation and emission properties spanning wavelengths from approximately 540 to 715 nm. This spectral diversity enables multicolor imaging approaches (Plamont et al., 2016; Tebo et al., 2021). Importantly, the reversible nature of FAST labeling allows fluorogen addition on demand, enhancing experimental flexibility. Overall, FAST tags are designed to improve fluorescence brightness and specificity while minimizing background signal, thereby enabling sensitive visualization of biological structures.

In addition, analogous to Split‐GFP systems used to study protein–protein interactions (Romei & Boxer, 2019), several Split‐FAST variants have been developed for similar applications (Rakotoarison et al., 2024; Tebo et al., 2021; Tebo & Gautier, 2019). Compared with Split‐GFP, Split‐FAST offers the advantage of reversibility and features fragments with markedly different sizes, including a minimal C‐terminal fragment of only 10 or 11 amino acids, depending on the variant. One of the most recent versions, derived from a PYP ortholog identified in *Rheinheimera* sp. A13L (RspA), termed Split RspA‐FAST, exhibits enhanced sensitivity for detecting protein–protein interactions. Moreover, it allows modulation of fluorescence emission colors (yellow, green, or red) through the use of distinct fluorogens, providing additional experimental versatility (Rakotoarison et al., 2024).

To date, FAST proteins have been successfully applied in prokaryotes, yeast, and animal systems, demonstrating their broad utility in cell biology. In this study, we investigate the feasibility of adapting the FAST system for use in plants and evaluate the potential advantages of this novel fluorescent reporter for plant cell biology.

## RESULTS

### FAST proteins are functional in Arabidopsis

The Green‐ and Red‐FAST proteins (Figure 1a) were cloned under the control of the constitutive promoter cauliflower mosaic virus 35S doubled (p35S^2^) into a binary vector to generate and select homozygous transgenic Arabidopsis seedlings constitutively expressing these proteins (Amack & Antunes, 2020). The corresponding fluorogens, Lime for Green‐FAST and Coral for Red‐FAST, were supplied to the roots of transgenic plants at a final concentration of 15 μM. A fluorescence signal was detected in the Arabidopsis root by confocal microscopy following the fluorogen application (Figure 1b). The fluorescence signal corresponded to the expected cytosolic and nuclear localization of the FAST proteins. Owing to their small size, these proteins can readily diffuse into the nucleus, similar to GFP (Haseloff et al., 1997). As described previously (Tebo et al., 2021), optimal excitation of Green‐FAST occurred at 488 nm. Excitation at 514 nm was also possible but resulted in approximately a twofold reduction in the intensity (Figure 1c), with a maximal emission peak at 540 nm. Spectral analysis of Red‐FAST revealed a more complex emission profile, with a major peak at 605 nm and a secondary peak around 665 nm, which could be resolved using 514‐nm excitation (Figure 1c, Figure S1). Notably, excitation at 561 nm produced a substantially stronger emission signal, detectable over a broader collection window (575–700 nm; Figure 1c, Figure S1), similar to that described in animal cells (Tebo et al., 2021). While root fluorescence was detectable within seconds following fluorogen addition, staining of aerial tissues (hypocotyl, Figure S2a,b, and leaves) was not observed within this timeframe. We therefore investigated the effect of seedling incubation time in the fluorogen solution (Figure 1d–g and S2a,b) and observed an optimal fluorescence emission signal, which doubled for roots incubated with the Lime fluorogen for 30–35 min. In contrast, the Coral fluorogen appeared more permeant through cellular barriers, as incubation time had no significant effect on the fluorescence signal in the roots (Figure 1d). Both fluorogens readily penetrated root tissues, enabling the labeling of the innermost cell layers (Figure S3).

Next, we examined the effect of fluorogens concentration (Figure 1e). The fluorescence signal increased for both Green‐FAST and Red‐FAST in accordance with the amount of their respective fluorogens. Green‐FAST responded to a lower Lime fluorogen concentration (1 μM), a level at which Red‐FAST did not produce a clear signal with the Coral fluorogen (Figure 1e). Moreover, Green‐FAST exhibited a more limited dynamic range in response to increasing fluorogen concentrations (threefold) compared with Red‐FAST (12–15‐fold). Most subsequent experiments were performed using 10–15 μM of fluorogens, which provided a favorable balance between signal efficiency and fluorogen usage. Notably, after optimizing incubation time (35–40 min) and fluorogen concentration (10–15 μM), fluorescence from Green‐ and Red‐FAST could also be clearly detected in the aerial parts of the seedlings, including hypocotyls and leaves (Figure 1f–g, Figures S2a,b and S4), with emission spectra similar to those observed in roots (Figures S1c,d and S2c). It should be noted that the secondary peak around 680 nm in the Green‐FAST spectra in leaves corresponds to fluorescence from chloroplasts due to chlorophyll and carotenoids superimposed on the signal from nuclei in the stack analyzed (Figure S2c). Therefore, when imaging tissues containing chloroplasts, a detection window below 650 nm is recommended to avoid overlap with chloroplast autofluorescence (Figure S4).

Various controls on leaves and roots clearly indicate that fluorogens do not exhibit any fluorescence per se when introduced into a wild‐type plant (Figure S4). Control experiments performed on leaves and roots clearly demonstrated that no fluorescence signal was detected from the fluorogens alone when incubated into wild‐type plants, nor from Green‐FAST or Red‐FAST proteins in transgenic plants in the absence of their respective Lime or Coral fluorogens (Figure S4). These controls also confirmed the specificity of the fluorescence signals observed with the Coral/Red‐FAST and Lime/Green‐FAST pairs (Figures S4 and S5). Indeed, the mismatched combinations Coral/Green‐FAST and Lime/Red‐FAST did not produce any significant fluorescence signal at 488 nm and 561 nm, the optimal excitation wavelengths used respectively for Green‐FAST and Red‐FAST (Figure S5). Figure S4 further demonstrates that the conditions selected for the fluorescence signal associated to Red‐ and Green‐FAST proteins with their respective fluorogens do not overlap with chlorophyll fluorescence signal.

We then evaluated fluorogens' toxicity in both the absence (WT) and presence of their respective FAST partners. Plants (Col, Red‐FAST, or Green‐FAST) were incubated under sterile conditions in dimethyl sulfoxide, with or without Coral or Lime fluorogens (15 μM) for 35 min, and subsequently returned to Petri dishes for root growth analysis. Because root growth is highly sensitive to environmental perturbations (Balzergue et al., 2017; Mercier et al., 2021; Sarrobert et al., 2000), it provides a robust proxy for toxicity assessment. Daily root elongation was measured 5–7 days post treatment to allow recovery from dimethyl sulfoxide exposure. No toxicity was detected for Lime or Coral fluorogens, either in wild‐type or in transgenic plants expressing their cognate FAST partners (Figure S6).

### Interest of FAST‐specific features compared to commonly used fluorescent proteins

**Figure 2:**
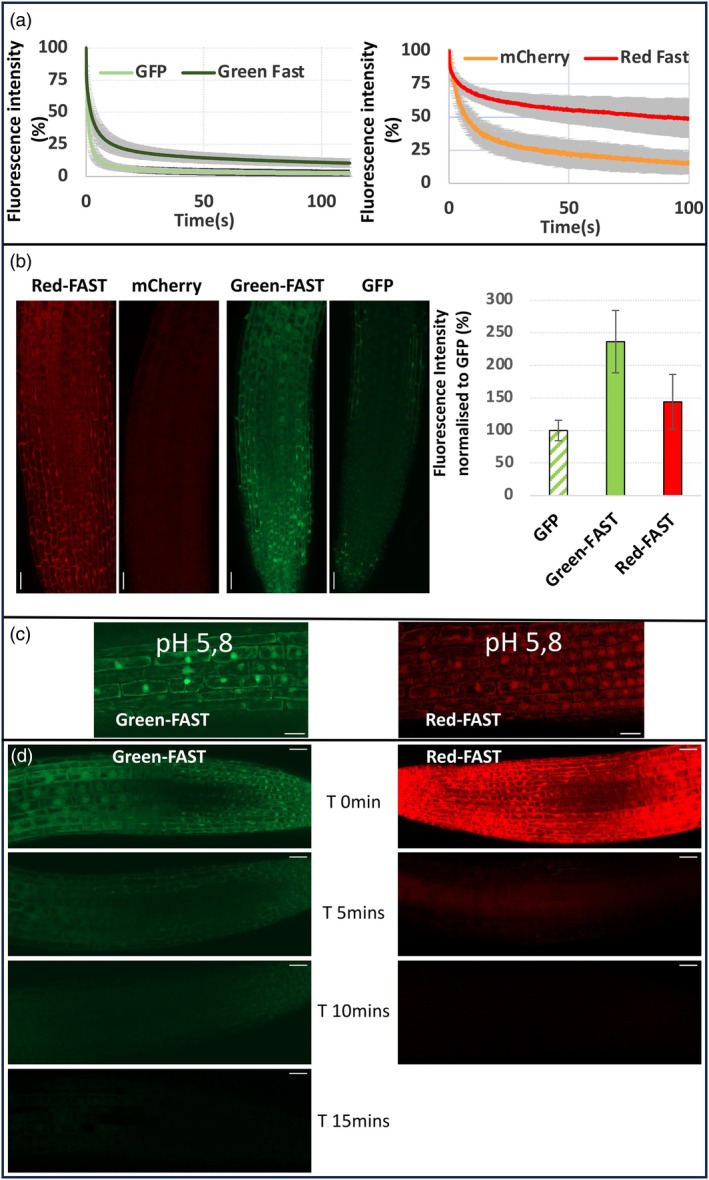
Analysis of some features of Green‐ and Red‐FAST constitutively expressed by 35S promoter fusion in plants. (a) Comparison between the bleaching of the Green‐FAST protein and eGFP (488 nm, 0.46 mW) or Red‐FAST and mCherry (561 nm, 0.16 mW). Plantlets were incubated 35 min in fluorogens before imaging. For each condition, 15 nuclei were imaged with 3 ROI per nucleus defined for quantification. The fluorescence intensity at t = 0 was normalized to 100%. (b) Fluorescence signals of Green‐FAST; eGFP; Red‐FAST and mCherry recorded under identical imaging conditions (laser intensity, Z‐stack parameters and detector gain). On the right, the graph compares the fluorescence intensity of Green‐FAST and Red‐FAST (in the presence of their respective fluorogens) to that of eGFP, with normalization to laser power and transcript levels of Green‐FAST, Red‐FAST, and eGFP. Three seedlings with 5 ROI each are used for mean and standard deviation measurements. (c) Imaging Red‐ or Green‐FAST proteins after fixation in paraformaldehyde (PFA) 4% (w/v) buffered at pH 5.8. (d) Washing incubation time required to abolish the fluorescence signal from Green‐ and Red‐FAST after 2 min incubation with 15 μM of the Lime or Coral fluorogen, respectively, in roots. Scale bar: 25 μm. The error bars on the graphs represent the standard deviations.

Photobleaching, the irreversible degradation of a fluorophore under excitation light, is often a limiting factor in microscopy. We therefore assessed the photobleaching of Green‐FAST and Red‐FAST proteins *in planta*. Compared with eGFP and mCherry, the FAST proteins proved more resistant to photobleaching, albeit to varying degrees (Figure 2a). The Green‐FAST retains 10 to 15% activity over more than 50 sec, whereas the eGFP signal was no longer detected (Figure 2a left). Interestingly, under red excitation (561 nm), the Red‐FAST protein exhibited approximately three‐fold greater resistance than mCherry (Figure 2a right). The decay curves fitted a decreasing exponential model (calculated with the PRISM software). This allowed us to determine the half‐life of eGFP (1.36 sec), Green‐FAST (5.29 sec), mCherry (8.17 sec), and Red‐FAST (17.03 sec), confirming the superior photostability of FAST proteins.

Another important feature is the fluorescence intensity of FAST proteins compared with eGFP. After normalization for laser power and transcript levels of Green‐FAST, Red‐FAST, and eGFP (relative to actin), Green‐FAST was found to be more than twice as bright (×2.4) compared to eGFP, whereas Red‐FAST was about 40% brighter (Figure 2b).

Cell biology experiments often require sample fixation in acidic buffer, a treatment known to alter proteins and frequently reduce or abolish GFP fluorescence (Figure S7). Both Green‐ and Red‐FAST (Figure 2c) present in the nuclei remained resistant to fixation, retaining their ability to bind fluorogens and produce a clear fluorescence signal at pH 5.8, the standard condition in our protocol (Hani et al., 2021). Even at more acidic pH (pH 5), both FAST proteins maintained detectable fluorescence, whereas GFP fluorescence was strongly impaired, leading to loss of nuclear labeling (Figure S7). Notably, under these conditions, Coral/Red‐FAST fluorescence remained unaffected, while Lime/Green‐FAST fluorescence was partially reduced.

A further advantage of FAST proteins is the reversibility of fluorogen binding. The fluorescence signal can be removed by washing the sample (Figure 2d), with the required wash duration depending on both the incubation time and the fluorogen used. For instance, a 1‐ to 2‐min incubation, which is sufficient for effective Red‐FAST labeling with Coral fluorogen (Figure 1e), requires only 5–10 min of washing to eliminate the signal. In contrast, Lime fluorogen, which is less permeant (Figure 1e), necessitates an extended wash of approximately 15 min (Figure 2d).

### FAST proteins are good reporter genes for *in vivo* labeling

**Figure 3:**
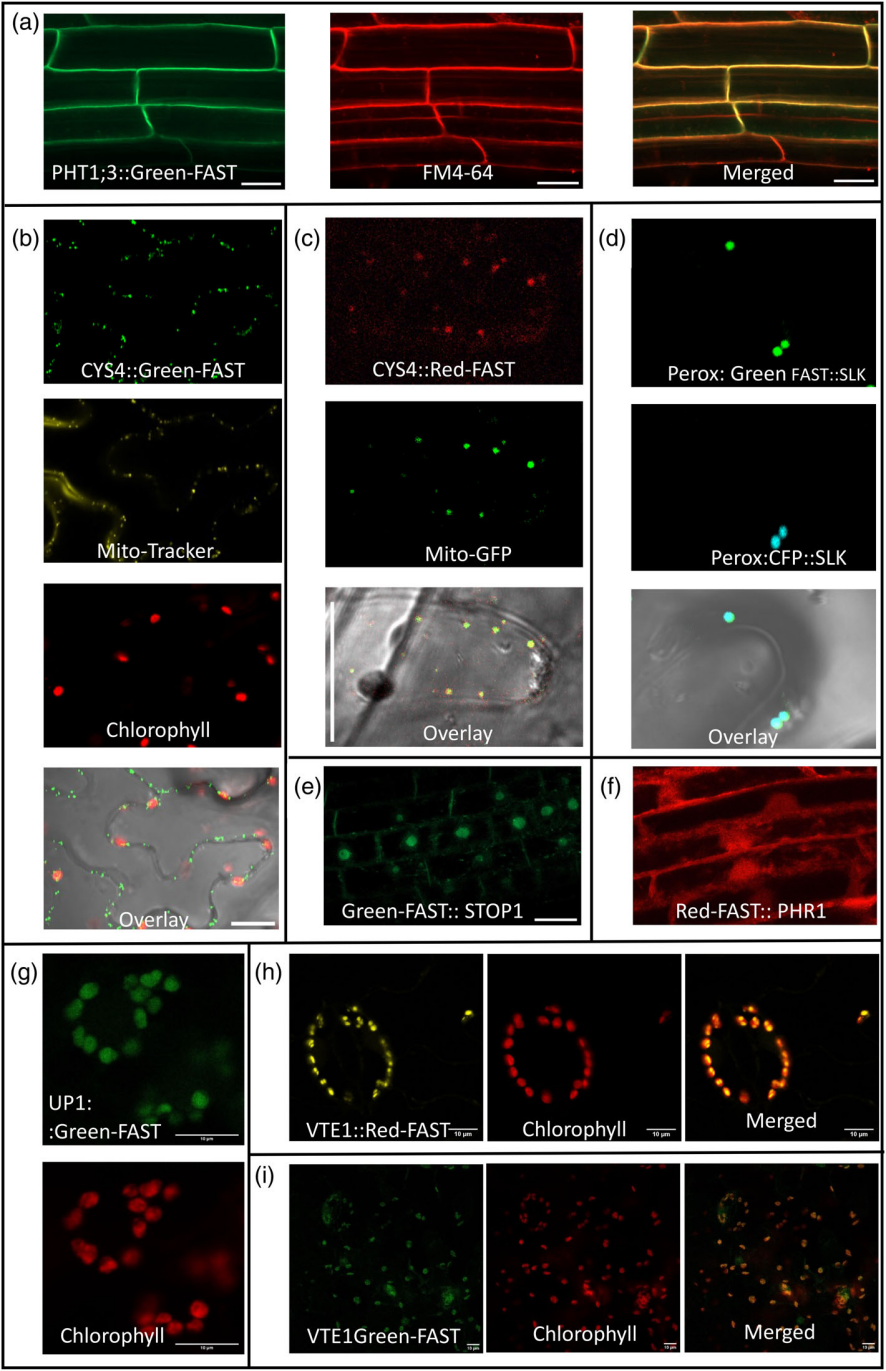
FAST proteins can be used for labeling different cellular compartments. (a) Plasma membrane visualization in roots (labeled by FM4‐64 marker) with a fusion between Green‐FAST and the phosphate transporter PHT1;3 (At5g43360). (b) Mitochondria visualization in epidermal leaf cells (labeled by MitoTracker Orange) with a fusion between the matrix protein CYS4 (At4g16500) and Green‐FAST. (c) Mitochondria visualization in epidermal leaf cells (labeled by Mito‐GFP) with a fusion between CYS4 (At4g16500) and Red‐FAST. (d) Peroxisome targeting peptide “SLK” fused to the CFP or to the Green‐FAST visualized in epidermal leaf cells. (e) Nuclei labeling with Green‐FAST fused to STOP1 (At1g34370) transcription factor in roots. (f) Nuclei labeling with Red‐FAST fused to PHR1 transcription factor (At4g28610) in roots. (g) Chloroplast (visualized by chlorophyll autofluorescence Laser 633 nm 0.33% Detector gain 650–750 nm) envelope labeling with UP1 (At1g11320) fusion to Green‐FAST in leaves (Laser 488 nm 0.6% Detector gain 800 V Emission spectra 500–550 nm). (h, i) Chloroplast (visualized by chlorophyll autofluorescence) stroma labeling with VTE1 (At4g32770) fusion with Green‐ or Red‐FAST proteins (Laser 488 nm 0.6% Detector gain 800 V Emission spectra 500–550 nm for Green‐FAST or Laser 514 nm 0.6% Detector gain 800 V Emission spectra 570–630 nm). Appropriate fluorogen (Coral or Lime, 10 μM) were used. Samples observed were obtained by stable (a, e, f) or transient (b, c, d, g, h, i) transformation in *Arabidopsis thaliana* or *Nicotiana benthamiana*, respectively.

The small size of FAST proteins (and their specific properties described above) makes them excellent candidates as reporter genes for localizing proteins in plants. To demonstrate this potential, we tested several candidate proteins *via* stable transformation (Figure 3a,e,f) or transient expression in *Nicotiana benthamiana* leaves (Figure 3b–d,g–i), a widely used system for rapid protein localization. We successfully labeled various membrane proteins, including plasma membrane protein; Figure 3a and UP1 (Bouchnak et al., 2019) in the chloroplast envelope (Figure 3g). FAST also enabled labeling of distinct cellular compartments: the mitochondrial matrix with CYS4‐FAST (Figure 3b,c), peroxisomes via a C‐terminal peroxisomal targeting “SLK” motif (Figure 3d), and the nucleus (Figure 3e,f) with FAST‐PHR1 or FAST‐STOP1 transcription factors (Godon et al., 2019; Nussaume & Kanno, 2024; Rubio et al., 2001). For chloroplasts, FAST labeled both the envelope (UP1; Figure 3g) and internal compartments such as the stroma using VTE1 (Bouchnak et al., 2019; Figure 3h,i).

### Split‐FAST proteins can monitor protein–protein interactions

**Figure 4:**
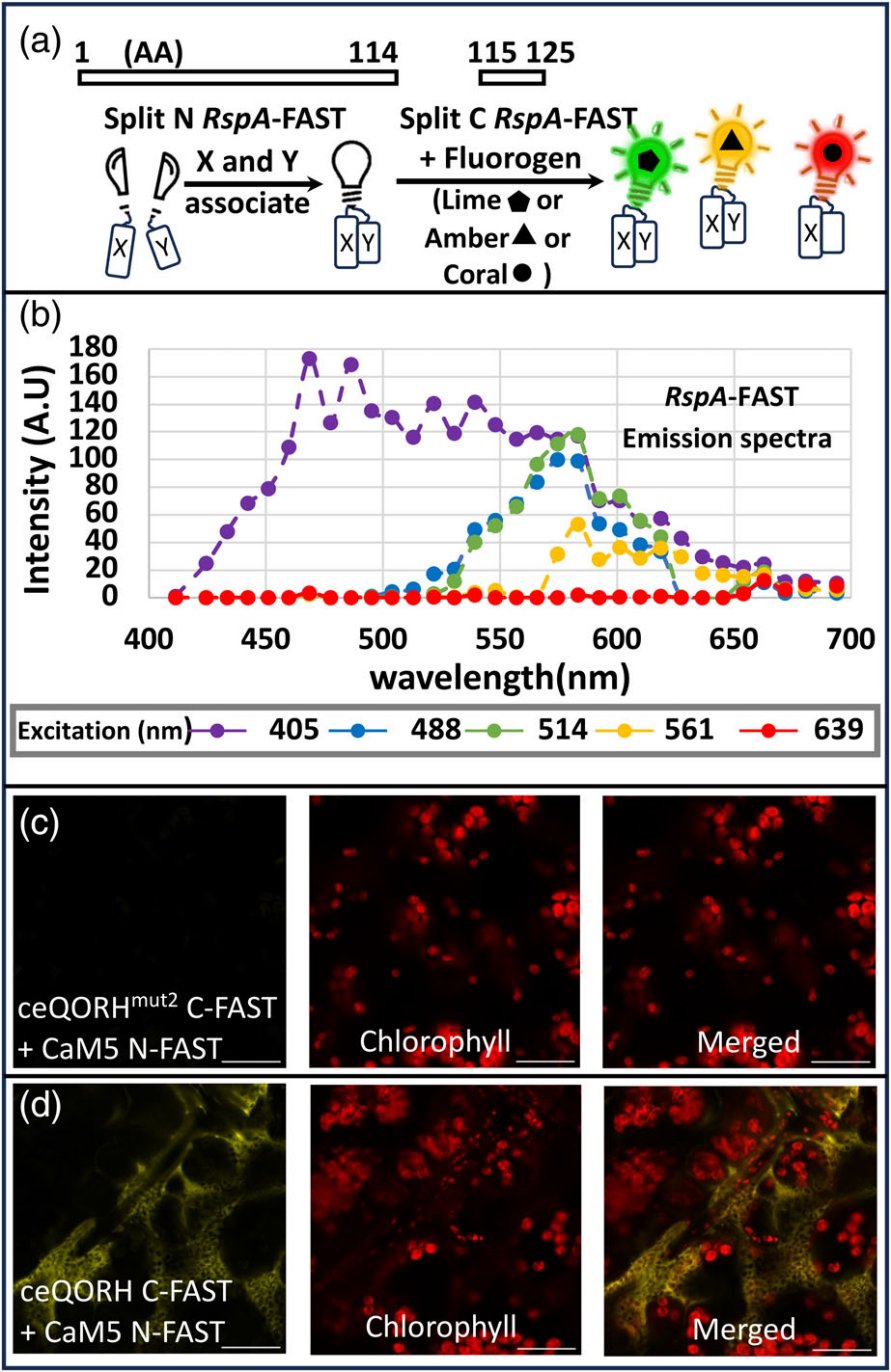
Protein–Protein interactions can be visualized by Split *RspA*‐FAST system. (a) Diagram illustrating the Split *RspA*‐FAST principle with the different fluorogens that can be used: Lime, Coral or Amber. (b) Emission spectra of Split *RspA*‐FAST in response to different excitation when combined with Amber fluorogen. (c) Negative control showing absence of interaction between CAM5::*RspA* N‐FAST and ceQORH^mut2^ *RspA* C‐FAST (Laser 488 nm 2% Detector gain 850 V Emission spectra 530–670 nm) in the presence of Amber fluorogen (1 μM). (d) The fluorescence signal, promoted by CAM5 *RspA::*N‐FAST and ceQORH::*RspA* C‐FAST interactions, is observed in the presence of Amber (1 μM) fluorogen (left image). For (c, d) an overlay (right image) with chlorophyll (middle image) is provided. CAM5 and ceQORH are encoded respectively by At4g13010 and At2g27030.

Different variants of FAST proteins have been developed to study *in vivo* protein–protein interactions (PPIs). One of the most advanced is Split RspA‐FAST (Rakotoarison et al., 2024). This system (Figure 4a) exhibits lower self‐complementation, higher brightness, and an increased dynamic range compared to the previously published Split‐FAST system, making it optimal for monitoring PPIs (Rakotoarison et al., 2024). Split RspA‐FAST consists of two fragments (114 and 11 amino acids, respectively) that, like Split GFP, must be fused to the proteins of interest. Reconstitution of the full RspA‐FAST indicates a physical interaction between the two proteins of interest.

A notable feature of RspA‐FAST is its compatibility with a broad spectrum of fluorogens, including Coral, Lime, and Amber. Figure 4b and Figure S8a show the emission spectra of these fluorogens. For Amber, excitation at 488 or 514 nm produced emission peaks at 575 nm and 583 nm, respectively (Figure 4b). Lime and Coral, excited at the same wavelengths, exhibited very similar fluorescence profiles (Figure S8a), with emission peaks at 566/575 nm for Lime and 583/592 nm for Coral. Interestingly, Coral combined with RspA‐FAST could also be excited at 561 nm, yielding a twofold weaker but detectable signal at 601 nm (Figure S8a). This flexibility allows optimization of excitation sources and emission windows according to other markers used in experiments.

The ability of the Split‐FAST system in plants to assess PPI was tested by fusing the N‐terminal RspA‐FAST fragment to the calmodulin isoform CAM5 and the C‐terminal fragment to the NADPH‐dependent α, β‐unsaturated oxoene reductase ceQORH (Curien et al., 2016). Previous studies using bimolecular fluorescence complementation and reverse genetics showed that the CAM5–ceQORH complex is retained in the cytosol (Moyet et al., 2019). To confirm signal specificity, each fusion was first tested separately with the Amber fluorogen, and no fluorescence was detected (Figure S8b). Interaction between ceQORH and CAM5 depends on the C‐terminal region (residues 255–277) of ceQORH (Moyet et al., 2019). As a negative control, a mutated ceQORH (ceQORHmut2), which cannot interact with CAM5, was used. Co‐expression of ceQORHmut2::C‐FAST with CAM5::N‐FAST in *N. benthamiana* leaves produced no fluorescence (Figure 4c), whereas co‐expression with wild‐type ceQORH resulted in a clear cytosolic fluorescence signal after Amber addition (Figure 4d). Similar results were obtained using the Lime fluorogen, demonstrating the versatility and reliability of the Split‐FAST system for detecting PPIs in plants (Figure S8c).

## DISCUSSION

Cell biology has undergone rapid development in recent years, and bioimaging faced a flood of fluorescent proteins (Shaner et al., 2005). In this study, we evaluated the applicability of FAST and its derivatives in plants, a novel system for visualizing protein localization and protein–protein interactions. Notably, these proteins can be switched on or off at will by adding or removing the fluorogens. This feature enables highly specific observation of protein localization by subtracting the nonspecific fluorescence signal detected prior to fluorogen addition.

*In vivo*, the fluorogens penetrate Arabidopsis roots almost immediately, reaching even the innermost cell layers. Leaf penetration takes slightly longer (15–30 min) but does not require any special additional treatment. This delay likely reflects the protective wax layers (cuticle) covering aerial organs, which limit direct fluorogen penetration. Furthermore, the reduced phloem flow associated with the low transpiration rates of *in vitro*–grown plants, as in this study, probably further restricts the fluorogen uptake and their systemic distribution from the roots, necessitating longer incubation times for aerial tissues.

Therefore, FAST proteins can be used to study all seedling tissues under physiological conditions. Green‐ and Red‐FAST proteins each bind a single fluorogen, producing distinct responses. This specificity was achieved through site‐directed mutagenesis to selectively target coral or lime fluorogens for Red‐FAST and Green‐FAST, respectively (Tebo et al., 2021). This also opens the possibility of combining them in future experiments, as previously demonstrated in animal systems (Tebo et al., 2021).

Both proteins exhibit higher photostability than classical reporters such as eGFP or mCherry. This improvement is particularly significant for Red‐FAST: although mCherry is widely used as a red fluorescent reporter, it is threefold less resistant to photobleaching, highlighting Red‐FAST as a notable advancement in the field.

RspA‐FAST offers remarkable versatility, as it can emit distinct fluorescence signals (green, yellow, or red) depending on the fluorogen applied. Consequently, a single transgenic line can be combined with a broad range of fluorescent markers or dyes as required. This feature is likely to be particularly attractive for users aiming to image multiple proteins simultaneously. Moreover, its relatively broad excitation spectrum provides additional flexibility, facilitating combination with various cellular markers or multiple GFP variants.

FAST proteins can be targeted to different cellular compartments and remain functional across a range of pH conditions, including neutral pH in the nucleus and alkaline environments such as peroxisomes and mitochondria (Shen et al., 2013). Our fixation experiments further indicate that FAST proteins exhibit greater resistance to acidic conditions (pH 5; Figure S7) than conventional eGFP. Further investigations will be necessary to determine whether this acid stability permits their use in highly acidic compartments, such as vacuoles or autophagosomes (Shen et al., 2013), as has been reported for specific GFP variants (Shinoda et al., 2018).

Compared with GFP‐like proteins, FAST proteins offer several intrinsic advantages. First, they are approximately half the size (125 amino acids, 14 kDa; (Tebo et al., 2021)), thereby reducing the risk of functional interference when fused to proteins of interest. This advantage is particularly relevant for the analysis of PPIs. In the Split‐FAST system, the protein is divided into two highly unequal fragments, one consisting of only 10–11 amino acids (Tebo & Gautier, 2019), which minimizes steric hindrance and functional perturbation for at least one interaction partner. By contrast, in Split‐GFP systems, the smallest fragment typically comprises 16 amino acids (Feng et al., 2017).

In addition, FAST proteins remain fully monomeric at concentrations up to the millimolar range (Plamont et al., 2016), whereas many GFP‐like proteins have a tendency to dimerize (Shaner et al., 2005). Although GFP‐derived reporters are known for their high stability, sometimes even stabilizing the proteins to which they are fused (Janczak et al., 2015), FAST appears to limit such stabilization artifacts (Rakotoarison et al., 2024).

The RspA‐FAST variant used in this study represents the latest generation of PYP orthologs developed in animal systems, engineered for increased brightness and sensitivity (Rakotoarison et al., 2024). Notably, it also displays enhanced dynamic properties and Split‐FAST has been successfully employed in animal models to monitor the dynamics of protein complexes in real time (Rakotoarison et al., 2024). This capability would constitute a major advance for plant cell biology compared with Split‐GFP, which forms highly stable and essentially irreversible complexes upon protein association. Split‐FAST may also provide an attractive alternative to FRET‐based approaches, which are technically demanding and potentially prone to artifacts related to the size and stability of GFP derivatives (Bayle et al., 2008).

This dynamic aspect, not explored in the present publication (parameters affecting CAM5/ceQORH interaction are ignored) will be studied in the near future with other protein combinations. Given the efficient transfer of FAST technology to plant systems demonstrated here, we are optimistic about its successful implementation. Importantly, Split‐FAST is not restricted to binary interactions: tripartite interactions can also be monitored either by dividing Split‐FAST into three fragments (Rakotoarison et al., 2024) or by combining the C‐termini of Green‐ and Red‐FAST with their shared N‐terminal fragment (Tebo et al., 2021). This versatility opens exciting new perspectives for plant cell biology.

## EXPERIMENTAL PROCEDURES

### Cloning

#### For constitutive expression of Green‐FAST or Red‐FAST

The Green and Red‐FAST proteins were amplified by PCR using pAG460‐FRB‐greenNFAST (plasmid Addgene #160361) or pAG461‐FRB‐redNFAST (plasmid Addgene #160362) as matrix and oligonucleotides (ATGGAGCATGTTGCCTTTGG Fw and CAATAGCTGTCACCGGAAAGGGCTTTCTTCATGTG Rv). The missing C‐FAST‐11 end (coding for GDSYWVFVKRV) was introduced by adding the corresponding 5′ extension at the end of a PCR oligonucleotide (CACCCGTTTCACAAAGACCCAATAGCTGTCACC Rv) used to reamplify the FAST protein with (ATGGAGCATGTTGCCTTTGG Fw). The amplified fragment was cloned into pGEM^®^‐T easy vector (Promega) for amplification. Two restriction sites (*Hind*III and *Xba*I) were also introduced at each extremity of these oligonucleotides (AAGCTTATGGAGCATGTTGCCTTTGG Fw and TCTAGACTACACCCGTTTCACAAAGACCC Rv) for their cloning in multicloning site of pBSK located in front of the p35S^2^ promoter present in the pKYLX71/pBSK (Chu et al., 2018; Schardl et al., 1987). The cassette containing the 35S^2^ promoter fused to the FAST protein was recovered by *Eco*RI and *Cla*I digestion and introduced at the same restriction sites in the binary vector pGreen II‐0179 (Hellens et al., 2005). This vector is derived from pGreenII 0000 by insertion of a 35S‐hyg cassette into the *Hpa*I restriction site of the T‐DNA Left Border. This offers selection on hygromycin for the identification of plant transformants.

#### For constitutive expression of cellular compartments genes

##### For nuclear markers

Two constructs were built using the Golden Gate cloning technique (Weber et al., 2011). The level0 vectors pICH51266 (Addgene plasmid #50267; http://n2t.net/addgene:50267; RRID:Addgene_50267) and pICH41414 (Addgene plasmid #50337; http://n2t.net/addgene:50337; RRID:Addgene_50337), containing respectively 35S promoter and terminator, were used. The Red‐ or Green‐FAST coding sequence with their linkers was optimized for plant codon usage, synthesized and cloned by Genewiz (Figure S9; https://www.genewiz.com), in level 0 vector pICH41258 (Addgene plasmid #47987; http://n2t.net/addgene:47987; RRID:Addgene_47987). These plasmids will be available from Addgene. For PHR1 (Rubio et al., 2001), coding sequence was synthesized and cloned by Genewiz in level 0 vector pICH 41264 (Addgene plasmid #47993; http://n2t.net/addgene:47993; RRID:Addgene_47993). For STOP1 (Balzergue et al., 2017), coding sequence without stop codon was synthesized and cloned by Genewiz in the pAGM1287 level 0 vector (Addgene plasmid #47996; http://n2t.net/addgene:47996; RRID:Addgene_47996) then amplified by PCR with oligonucleotides (GGCCGATTCATTAATCACTGAAGACTCAGGTATGGAAACTGAGGACGATTTGTGCAACACC Fw; CCACTGAAGAGCCACTTCGAAGACTCAAGCTTAGAGACTAGTATCTGAAACAGACTCACC Rv) to engineer the pICH41264 level 0 vector. The level 0 modules were assembled directly into a level 1 pICH86966 expression vector (Addgene plasmid # 48075; http://n2t.net/addgene:48075; RRID:Addgene_48075), creating a fusion between nuclear proteins and the FAST protein located at the N‐term. This allows the selection of plant transformants by kanamycin.

##### For plasma membrane marker

Level 0 vectors pICH51266 (Addgene plasmid #50267; http://n2t.net/addgene:50267; RRID:Addgene_50267) and pICH41414 (Addgene plasmid #50337; http://n2t.net/addgene:50337; RRID:Addgene_50337), containing respectively 35S Promoter and terminator were used. The PHT1;3 coding sequence without stop codon and *Bpi*I restriction site was synthesized and cloned by Genewiz in level 0 vector pAGM1287 (Figure S9). The Green‐FAST protein was amplified by PCR using as matrix pAG460‐FRB‐greenNFAST (plasmid Addgene #160361) and the oligonucleotides (TCCGGAGGAGGCGGCAGC Fw and GGAAAGGGCTTTCTTCATGTG Rv). The missing C‐FAST‐11 end (coding for GDSYWVFVKRV) was introduced by adding the corresponding 5′ extension at the end of a PCR oligonucleotide (CACCCGTTTCACAAAGACCCAATAGCTGTCACC Rv), used to reamplify the FAST protein with (TCCGGAGGAGGCGGCAGC Fw). The fragment containing the linker and Green‐FAST protein for C‐term fusion was PCR amplified with oligonucleotides (GTGTTCCGACGAATCGAAGACGGTTCGTCCGGAGGAGGCGGCAGC Fw; TTCTACCTCAGAAGACTTAAGCTCACACCCGTTTCACAAAGACCC Rv) and cloned in level 0 vector pAGM1301. The level 0 modules were assembled directly into a level 1 expression vector, pICH86966, allowing the selection of the plant transformants by kanamycin.

##### For plastidial marker

The coding regions of proteins UP1 and VTE1 were PCR‐amplified using two flanking primers, *Sal*I–N‐ter (GTCGACATGGACCCAATTGCTTCGG for UP1 and TCTGTCGACATGGAGATACGGAGCTTG for Vte1) and *Nco*I–C‐ter (CCATGGACAGCGACCAGTGAGACTTTAG for UP1 and ATCCCATGGACAGACCCGGTGGCTTG for VTE1). The PCR products were cloned into the pBlueScript SK^−^ vector (Stratagene). *Sal*I‐*Nco*I fragments cleaved from this plasmid were inserted into the *Sal*I–*Nco*I digested 35ΩpUC‐Green‐FAST or 35ΩpUC‐Red‐FAST to create the 35 Ω UP1/Vte1 Green‐FAST/Red‐FAST vectors (FAST proteins being located at the C‐term). From these constructs, the *EcoR*I–*Hind*III fragments were extracted and inserted into the *Eco*RI–*Hind*III–digested pEL103 (Moyet et al., 2019) binary vector (harboring kanamycin resistance for selection into plants).

##### For peroxisome and mitochondrial marker

All the cloning were performed using Gibson assembly (Gibson et al., 2009). The peroxisomal targeting sequence “SLK” motif was introduced at the *C*‐term end of the Green‐FAST sequence on synthesized oligonucleotides. The PCR were performed using the GreenFAST templates and the olionucleotides TTTGGAGAGGACACGCTGACAAGCTGACTATGGAGCATGTTGCCTTTGGCAG (Fw) and TGCCAAATGTTTGAACGATCTGCAGGCTAAAGCTTAGACACCCGTTTCACAAAGACCCA (rv). The mitochondrial marker was made by a fusion of the full‐length Cys4 matrix protein (At4g16500) with the Green‐FAST/Red‐FAST proteins located at the C‐term. Cys4 was amplified from *A. thaliana* cDNA using the oligonucleotides TTTGGAGAGGACACGCTGACAAGCTGACTATGGTGTTTTTCCGCAGCGTATC (Fw) and TCACTGCCAAAGGCAACATGCTCCATAGCAGATGAAGCTTTCTTACAATGGG (Rv). The Green‐FAST or Red‐FAST fragments were amplified using the corresponding DNA templates and the oligonucleotides AAGCCCATTGTAAGAAAGCTTCATCTGCTATGGAGCATGTTGCCTTTG GCAG (Fw) and TATTGCCAAATGTTTGAACGATCTGCAGGCTACACCCGTTTCACAAAGACCC (Rv). The PCR products were cloned in the *Sal*I and *Xba*I restriction sites present in the pFP108 vector (To et al., 2006).

#### For split‐FAST constructs

Synthetic genes (Figure S9) for ceQORH‐cFAST, Mut2_ceQORH‐cFAST and CaM5‐nFAST (Moyet et al., 2019) were purchased from Twist Bioscience (www.twistbioscience.com) and inserted directly into the plasmid pUC19‐GFP (Chiu et al., 1996) digested by *SalI–NotI*. This provided the opportunity to replace, under the control of the 35S promoter, the GFP present in the pUC plasmid by the synthetic gene of interest. From these constructs, the *Eco*RI–*Hind*III fragments were extracted and inserted into the *Eco*RI–*Hind*III–digested pEL103 binary vector (providing kanamycin resistance for the selection of transformed plants). All final constructs were verified by sequencing.

### Transient expression in *Nicotiana benthamiana*

Peroxisome and mitochondrial constructs were introduced into *Agrobacterium tumefaciens* GV3101:pMP90 strain (Koncz & Schell, 1986) whereas plastidial and split‐FAST constructs were transferred into C58 strains by heat shock transformation. Transgenic bacteria were selected on antibiotics. Mito‐GFP (CD3‐987) and perox‐CFP (CD3‐977) plasmids (Nelson et al., 2007) were retrieved from the NASC and used as mitochondrial and peroxisomal markers, respectively. The 10‐ml overnight cultures of *A. tumefaciens* were sedimented by a 10 min centrifugation at 4000 rpm and resuspended in 5 mL of Agroinfiltration buffer (1 mM MgCl_2_ + 200 μM acetosyringone). The OD at 600 nm was measured and the strains were diluted to obtain an OD600 nm of 1. The desired constructions (when co‐expression was required) were mixed 1:1 (v/v) and infiltrated with a 1‐mL syringe into 5 week‐old *Nicotiana benthamiana* leaves grown under a 16‐h light, 20°C/8‐h dark, 18°C cycle (light intensity 90 μmol/m^2^/s, humidity 70%). Silencing inhibitor P19 was always co‐infiltrated. Confocal imaging was performed 2 to 4 days after infiltration.

### Production of stable transformants

Transgenic lines produced by floral dipping (Harrison et al., 2006) were selected on Hoagland/2 media complemented with appropriate antibiotics (50 mg.L^−1^ kanamycin or hygromycin according to the binary vector used). The progenies, exhibiting 3:1 segregation, were carried to the T3 generation to identify homozygous lines carrying a single insertion locus.

### Plant materials and growth conditions

Wild‐type *A. thaliana* Col‐0 and *A. thaliana* seeds expressing all the different constructs were sterilized and grown vertically during 5 to 7 days on Hoagland medium diluted two‐fold in Petri dishes in a culture chamber under a 16‐h‐light/8‐h‐dark regime (25°C/22°C), as previously described (Sarrobert et al., 2000).

### Quantitative RT‐PCR

Total RNA was extracted from whole plantlets using the Direct‐Zol™ RNA MiniPrep (Zymo Research, Irvine, CA, USA, https://www.zymoresearch.com/) and treated with the RNase‐free DNase Set (Zymo Research) according to the manufacturer's instructions. Reverse transcription was performed on 500 ng of total RNA using Superscript vilo cdna synthesis kit (Invitrogen, Carlsbad, CA, USA, https://www.thermofisher.com/). Quantitative PCR was performed on a 480 LightCycler thermocycler (Roche Diagnostics GmbH, Mannhein, Germany, https://www.roche.com/) using the manufacturer's instructions with Light Cycler 480 SYBR Green I Master (Roche). For eGFP, primers give an amplicon of 81 bp (ACGACTTCTTCAAGTCCGCC Fw; TCTTGTAGTTGCCGTCGTCC Rv). For Green and Red‐FAST, primers give an amplicon of 109 bp (GGAGCATGTTGCCTTTGGCAG Fw; CCCGTCACCATCGAGCTGAAT Rv). The Actin2 gene (At3g18780) was used as a reference gene for normalization and detected using the oligonucleotides: GTCGTACAACCGGTATTGTGCTG Fw and CCTCTCTCTGTAAGGATCTTCATGAG Rv. The efficiency of the primers was tested using a dilution range of their respective targets showing a common value of two.

### Confocal imaging

Laser scanning confocal microscopy was performed on a microscope Zeiss LSM880 or LSM980 Airyscan2 with oil‐immersed (63×/1.4 and 40×/1.4), water‐immersed (40×/1.2) or dry 20×/0.8 DIC M27 Plan‐Apochromat objectives. Images were acquired using the ZEN software and processed in Fiji (ImageJ). The Lime, Amber and Coral fluorogens are available from The Twinkle Factory (https://www.the‐twinkle‐factory.com/fluorogens‐and‐molecular‐glues/) and correspond respectively to the following chemical identifiers HMBR, HBR‐3,5 DM, and HBR‐3,5DOM. They were diluted in a MgCl_2_ 1 mM solution and infiltrated with a syringe into the transformed *N. benthamiana* leaves just before confocal observation. They were used at 10 μM concentration for Lime or Coral. For experiments with *RspA* Split FAST, Lime and Amber were used at a 0.5 and 1 μM concentration respectively with a 15 min incubation time before observations. The MitoTracker™ Orange was used for mitochondria labeling at a final concentration of 250 nM added to the Lime solution before infiltration in leaves. MitoTracker™ Orange, Mito‐GFP, Perox‐CFP and Chlorophyll were respectively excited at 561 nm (1–2% laser capacity), 488 nm (10% laser capacity), 458 nm (10% laser capacity) and 633 nm (2% laser capacity). The fluorescence emission signal was collected respectively at 566–628 nm (600 V gain), 493–522 nm (800 V gain), 463–487 nm (800 V gain), and 647–721 nm (675 V gain). Multiple channel imaging was performed in sequential mode. For FAST excitation and emission, the details are provided in the result section.

As different wavelengths of excitation are possible for the different FAST proteins according to the dye chosen (see result part), such information is provided in the legend of the figures. For stable transformants, fluorogens were diluted in MS/10 liquid media (for concentration used refers to Figure legends). A 0‐ to 35‐min incubation period was performed before confocal observation. Green‐FAST, Red‐FAST, and Chlorophyll were respectively excited at 488 nm, 514 or 561 nm, and 639 nm (% laser capacity indicated on each figure). The fluorescence emission signal was collected respectively at 491–632 nm, 588–641 nm, and 654–713 nm (detector gain indicated with each figure).

For spectral acquisitions, the LSM Lambda mode with the 32 detectors of the confocal LSM980 Airyscan2 (each detector has a resolution of 10 nm) ranging from 411 until 694 nm with 8.8‐nm steps was used. We focused our analysis on nuclei images resulting from averaging four times the same scan (using bi‐directional scanning mode with a speed of four) to generate 16‐Bits/pixel images. The same regions of interest (ROI, calculated with the Unmix function provided by Zen Blue software from Zeiss) were conserved for the different laser excitations. The experiments were repeated in at least triplicate.

For bleaching experiments, images were acquired with the Confocal LSM980 Airyscan2 with the LD LCI Plan‐Apochromat 40X/1.2 Imm Korr DIC M27 objective. Nuclei were excited continuously with a 10% (0.46 mW) Laser for Green‐FAST and GFP and a 3% (0.16 mW) Laser for Red‐FAST and mCherry. Image acquisition was performed every 100 ms during 1000 cycles.

To compare fluorescence signal of Green‐FAST, Red‐FAST, and eGFP, 5 day‐old seedlings were incubated with their respective fluorogens during 35 min before imaging them with Plan‐Apochromat 20X/0.8 M27 objective. For each excitation, 488 nm (Green‐FAST/e‐GFP), 561 nm (Red‐FAST), a Z‐stack (13 μm) was imaged. Three seedlings with 5 ROI each were used for quantification of MIP images. For Green‐FAST/e‐GFP, laser 488 nm 0.2% Detector gain 750 V emission spectra 491–632 nm; for Red‐FAST, laser 561 nm 0.8% Detector gain 800 V emission spectra 588–641 nm. For each fluorescent protein, fluorescence intensity measured was normalized, taking into account laser intensity, its power, and the quantity of mRNA in plants. mRNA Green‐FAST, mRNA Red‐FAST, and mRNA GFP were quantified by quantitative RT‐PCR. Laser's power was measured with a power‐meter with Plan‐Apochromat 10X (Zeiss company) and was expressed in mW/spot, with a spot of 0.4356 μm^2^ (Figure S10).

To study reversibility of FAST staining, 5‐day‐old seedlings were put between slide and cover‐slip with 30 μL of fluorogens at 15 μM and immediately imaged with Plan‐Apochromat 20X/0.8 M27 objective. Acquisition of Z‐stack images to obtain a MIP of 19 μm for Green‐FAST and 36 μm for Red‐FAST. Plantlets were rinsed in 3 mL Hoagland/2 medium during 5, 10 or 15 min before imaging with the same Z‐stack. Laser 488 nm 0.2% Detector gain 750 V Emission spectra 491–632 nm for Green‐FAST; laser 561 nm 0.8% Detector gain 800 V emission spectra 588–641 nm for Red‐FAST. To test the specificity of fluorogens according Red‐ or Green‐FAST protein (Figures S4 and S5), 5‐day‐old seedlings were incubated in DMSO 0.3% with or without the fluorogens (15 μM) during 35 min before imaging them with Plan‐Apochromat 20X/0.8 M27 objective. For each excitation, 488 nm (Green‐FAST/e‐GFP), 561 nm (Red‐FAST), bright field, a Z‐stack (5 μm) is imaged.

### Toxicity tests

For the toxicity test, 5‐day‐old plantlets were incubated with or without Coral and Lime (final concentration 15 μM) in sterile Hoagland liquid medium diluted two‐fold, containing 0.3% DMSO. After 35 min, the plantlets were transferred onto sterile Petri dishes with two‐fold diluted Hoagland medium and returned to the culture chamber. After 5–7 days, root elongation was measured over a 24‐h period using the open‐source Fiji platform for biological image analysis and the NeuronJ plugin (Meijering et al., 2004; Schindelin et al., 2012).

### PFA fixation

5‐day‐old plantlets were fixed 20 min in 4% PFA diluted in MESX1 at pH 7 or 5 or 5.8 before incubation of 35 min in fluorogens at 15 μM. Confocal acquisition with Plan‐Apochromat 20X/0.8 M27 objective, laser 488 nm 0.8% Detector gain 850 for Green‐FAST and GFP, laser 561 nm 0.8% Detector gain 800 V for Red‐FAST.

## ACCESSION NUMBERS

ACT2 (At3g18780), CAM5 (At2g27030), CYS4 (At4g16500), ceQORH (At4g13010), PHR1 (At4g28610), PHT1; 3 (At5g43360), STOP1 (At1g34370), UP1 (At1g11320), VTE1 (At4g32770).

## AUTHOR CONTRIBUTIONS

PD, MM, LM, SP, JZ performed experiments and analyzed data. LN, BL, TD, EB, PD conceived the research and supervised the data analysis. LN and PD wrote the manuscript. AG provided the unpublished FAST protein and provided the fluorogens for the experiments. All authors commented on the manuscript and approved the content.

## CONFLICT OF INTEREST

AG is co‐founder and holds equity in Twinkle Bioscience/The Twinkle Factory, a company commercializing the FAST technology.

## Supporting information


**Figure S1.** Example of spectral images collected for Green‐(a, c) or Red‐FAST (b, d) proteins constitutively expressed by fusion with 35S promoter to produce the spectral analysis graphs from root of Figure 1 (for S1a,b) or leaves of Figure 2 (S1c,d).
**Figure S2.** (a) Vizualisation of the Green‐FAST (Laser 488 nm 0.2% Detector gain 750 V) proteins in the hypocotyl combined with chlorophyll fluorescence signal (laser 639 nm 2% Detector gain 700 V) following the addition of the fluorogen. (b) Vizualisation of the Red‐FAST proteins (Laser 561 nm 0.8% Detector gain 800 V) in the hypocotyl combined with chlorophyll fluorescence signal (laser 639 nm 2% Detector gain 700 V) just after addition of the fluorogen or 35 min later. (c) Emission spectra of Green‐FAST and Red‐FAST proteins in the hypocotyl as described in Figure 1. Scale bar: 25 μm. The different samples were imaged with a Plan‐Apochromat 20X/0.8 M27 objective. All FAST proteins are constitutively expressed by fusion with 35S promoter.
**Figure S3.** 3D fluorescence imaging of *Arabidopsis thaliana* roots expressing GFP, Green‐FAST, or Red‐FAST under the control of the CaMV 35S promoter. The FAST transformants were incubated (35 min) with the corresponding fluorogens prior to imaging. Three‐dimensional reconstructions and optical sections were generated using the 3D Viewer plugin in Fiji. YZ orthogonal views are shown. A total of 38, 41, and 43 optical sections were acquired for GFP‐, Green‐FAST–, and Red‐FAST–expressing transformants, respectively, with a z‐step of 1 μm. Scale bar for YZ orthogonal view: 20 μm.
**Figure S4.** Coral and Lime fluorogens do not exhibit detectable fluorescence in the absence of their corresponding FAST protein partner. Root and leaves fluorescence imaging of wild type and Red‐ or Green‐FAST transformants (previously incubated 35 min in solutions with or without the Coral and Lime fluorogens at 15 μM). Green‐FAST is detected at 488 nm with a 0.2% Laser. Signal is collected in a range of 491 to 632 nm with a digital gain of 750 V. Red‐FAST is detected at 561 nm with a 0.5% Laser. Signal is collected in a range of 588 to 641 nm with a digital gain of 800 V. Chlorophyll is detected at 639 nm with a 0.09% Laser and signal is collected in a range of 654 to 713 nm with a digital gain of 700 V. Scale bar: 20 μm.
**Figure S5.** Control experiments conducted in roots to assess the specificity of the Green‐FAST/Lime and Red‐FAST/Coral fluorogen–protein pairs. Samples were incubated 35 min in solutions with or without the Coral and Lime fluorogens at 15 μM before observation. Green‐FAST is detected at 488 nm with a 0.2% Laser. Signal is collected in a range of 491 to 632 nm with a digital gain of 750 V. Red‐FAST is detected at 561 nm with a 0.5% Laser. Signal is collected in a range of 588 to 641 nm with a digital gain of 800 V. Scale bar: 20 μm.
**Figure S6.** Toxicity analysis of Lime and Coral fluorogens on (a) wild type control (Col) or (b) transgenic Green‐ or Red‐FAST. Five days‐old plantlets were incubated 35 min in sterile Hoagland/2 liquid medium with DMSO 0.3% supplemented with or without Coral and Lime fluorogens (final concentration 15 μM) and then replanted (without rinsing) in Petri dishes with solid nutrient media before returning to culture chambers. After 5 (a) or 7 (b) days, root elongation was measured over 24 h. Results are provided in the two tables with the mean (measured over 10 samples) and the standard deviation. *t*‐test performed to compare treated and untreated plants revealed non‐significant (NS) differences.
**Figure S7.** Imaging GFP Red‐ or Green‐FAST proteins constitutively expressed by fusion with 35S promoter before or after fixation in paraformaldehyde 4% (w/v) (PFA) buffered at pH 5 or 7 during 20 min. Green‐FAST and GFP are detected at 488 nm with a 0.8% Laser with a digital gain of 850 V. Red‐FAST is detected at 561 nm with a 0.8% Laser with a digital gain of 800 V. The two magnified views at the top of the figure were adjusted using identical contrast settings to facilitate visualization of the fluorescent signal. Scale bar: 25 μm.
**Figure S8.** (a) Emission spectra of Split *RspA‐*FAST in response to different excitation when combined with Lime or Coral fluorogen. (b) Negative control showing absence of fluorescence with Amber (1 μM) for cells expressing only CAM5::*RspA* N‐FAST or ceQORH *RspA* C‐FAST. (c) Fluorescence signal promoted by CAM5::*RspA* N‐FAST and ceQORH::*RspA* C‐FAST interactions observed in the presence of Lime (0.5 μM) fluorogen (left image). CAM5 and ceQORH are encoded respectively by At4g13010 and At2g27030.
**Figure S9.** Synthetic genes synthesized by Twist Bioscience/Genewiz.
**Figure S10.** Measurements of the power of the lasers used expressed in mW/spot (spot of 0.4356 μm^2^ with Plan‐Apochromat 10X (Zeiss company) objective).

## Data Availability

The data that support the findings of this study are available upon request from the corresponding author. The data are not publicly available due to privacy or ethical restrictions.
