# Supplementary material for: The fluorescence‐activating and absorption‐shifting tag (FAST), a versatile protein marker for live plant cell imaging
Source: Plant J. 2026 Jun 12;126(5):e70966. doi: 10.1111/tpj.70966 (PMC13263057; doi:10.1111/tpj.70966)
Supplement: Supplementary file 1 — Figure S1. Example of spectral images collected for Green‐(a, c) or Red‐FAST (b, d) proteins constitutively expressed by fusion with 35S promoter to produce the spectral analysis graphs from root of Figure 1 (for S1a,b) or leaves of Figure 2 (S1c,d). Figure S2. (a) Vizualisation of the Green‐FAST (Laser 488 nm 0.2% Detector gain 750 V) proteins in the hypocotyl combined with chlorophyll fluorescence signal (laser 639 nm 2% Detector gain 700 V) following the addition of the fluorogen. (b) Vizualisation of the Red‐FAST proteins (Laser 561 nm 0.8% Detector gain 800 V) in the hypocotyl combined with chlorophyll fluorescence signal (laser 639 nm 2% Detector gain 700 V) just after addition of the fluorogen or 35 min later. (c) Emission spectra of Green‐FAST and Red‐FAST proteins in the hypocotyl as described in Figure 1. Scale bar: 25 μm. The different samples were imaged with a Plan‐Apochromat 20X/0.8 M27 objective. All FAST proteins are constitutively expressed by fusion with 35S promoter. Figure S3. 3D fluorescence imaging of Arabidopsis thaliana roots expressing GFP, Green‐FAST, or Red‐FAST under the control of the CaMV 35S promoter. The FAST transformants were incubated (35 min) with the corresponding fluorogens prior to imaging. Three‐dimensional reconstructions and optical sections were generated using the 3D Viewer plugin in Fiji. YZ orthogonal views are shown. A total of 38, 41, and 43 optical sections were acquired for GFP‐, Green‐FAST–, and Red‐FAST–expressing transformants, respectively, with a z‐step of 1 μm. Scale bar for YZ orthogonal view: 20 μm. Figure S4. Coral and Lime fluorogens do not exhibit detectable fluorescence in the absence of their corresponding FAST protein partner. Root and leaves fluorescence imaging of wild type and Red‐ or Green‐FAST transformants (previously incubated 35 min in solutions with or without the Coral and Lime fluorogens at 15 μM). Green‐FAST is detected at 488 nm with a 0.2% Laser. Signal is collected in a range of 491 to 63 [file TPJ-126-0-s001.pdf]

Figure S1

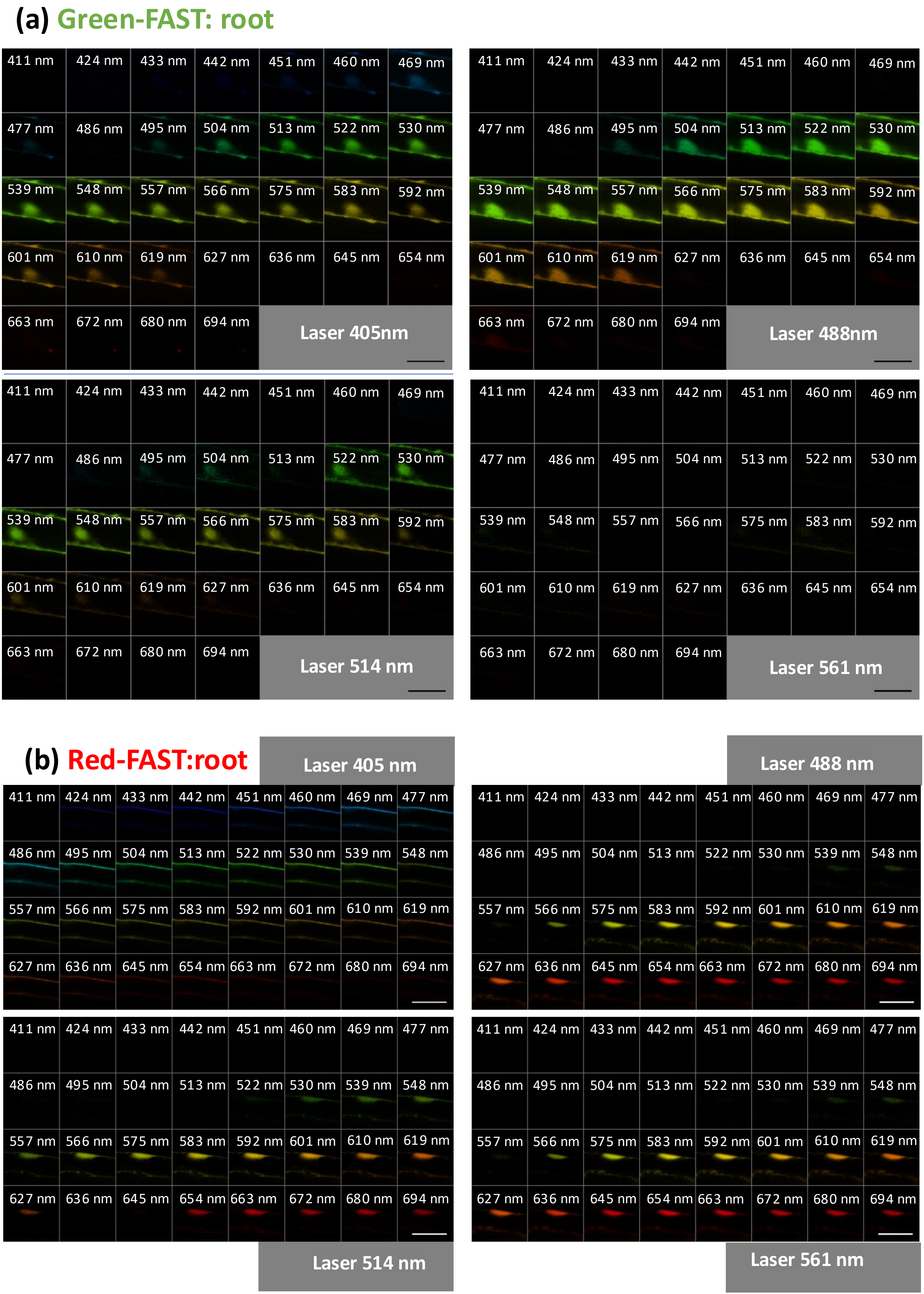

(a) Green-FAST: root
411 nm 424 nm 433 nm 442 nm 451 nm 460 nm 469 nm
477 nm 486 nm 495 nm 504 nm 513 nm 522 nm 530 nm
539 nm 548 nm 557 nm 566 nm 575 nm 583 nm 592 nm
601 nm 610 nm 619 nm 627 nm 636 nm 645 nm 654 nm
663 nm 672 nm 680 nm 694 nm
Laser 405nm
Laser 488nm
Laser 514 nm
Laser 561 nm
(b) Red-FAST:root
Laser 405 nm
Laser 488 nm
411 nm 424 nm 433 nm 442 nm 451 nm 460 nm 469 nm 477 nm
486 nm 495 nm 504 nm 513 nm 522 nm 530 nm 539 nm 548 nm
557 nm 566 nm 575 nm 583 nm 592 nm 601 nm 610 nm 619 nm
627 nm 636 nm 645 nm 654 nm 663 nm 672 nm 680 nm 694 nm
Laser 514 nm
Laser 561 nm

(c) Green-FAST leaves

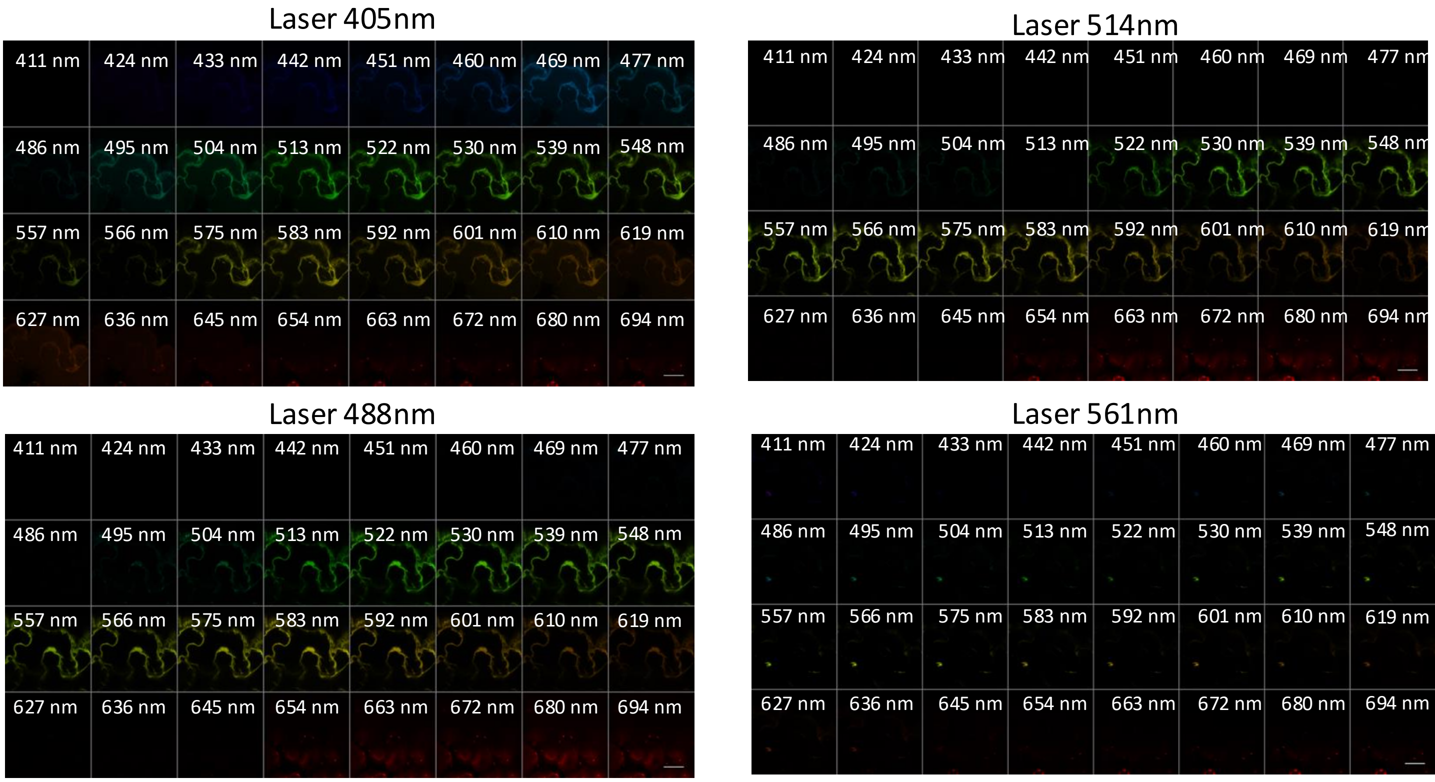

(d) Red-FAST leaves

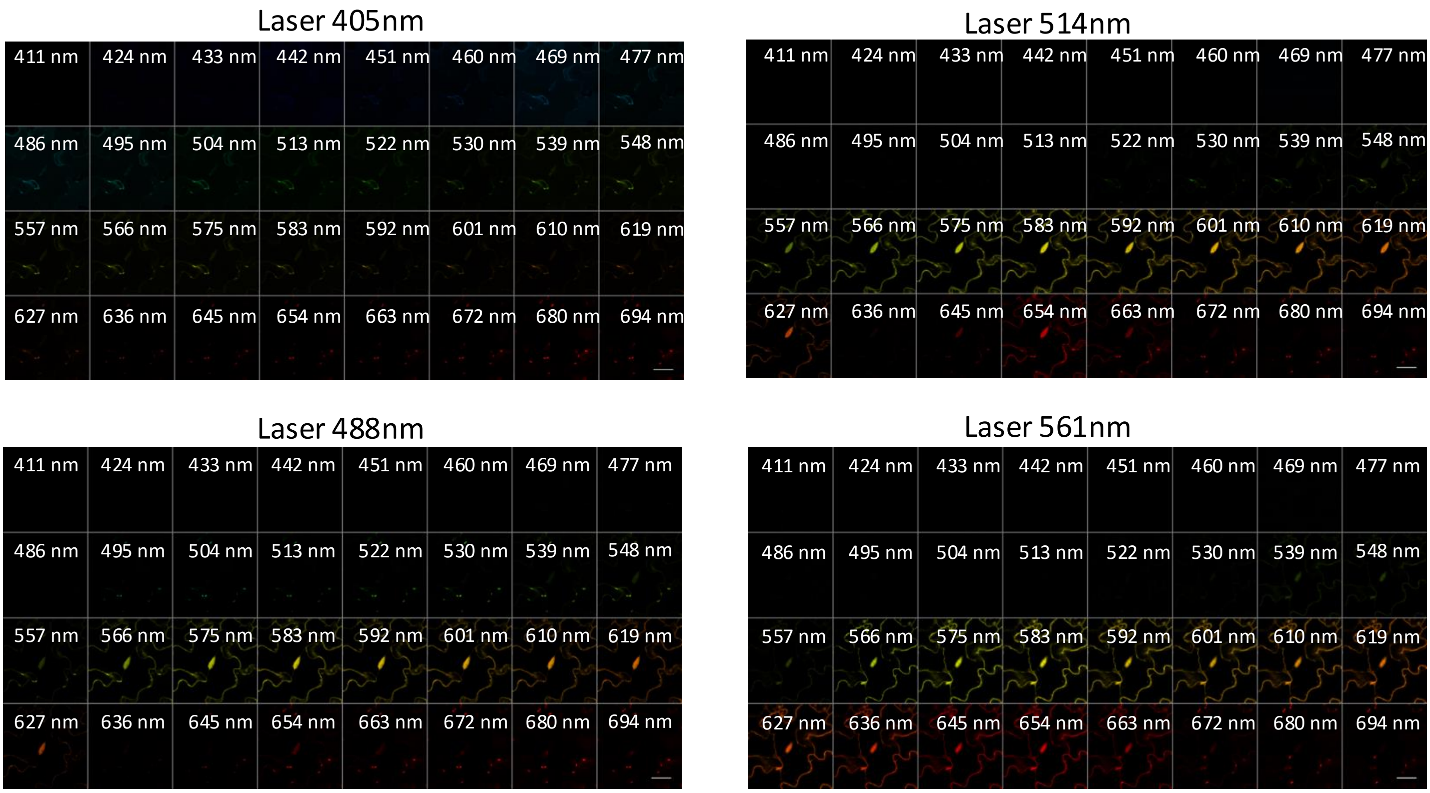

Figure S2

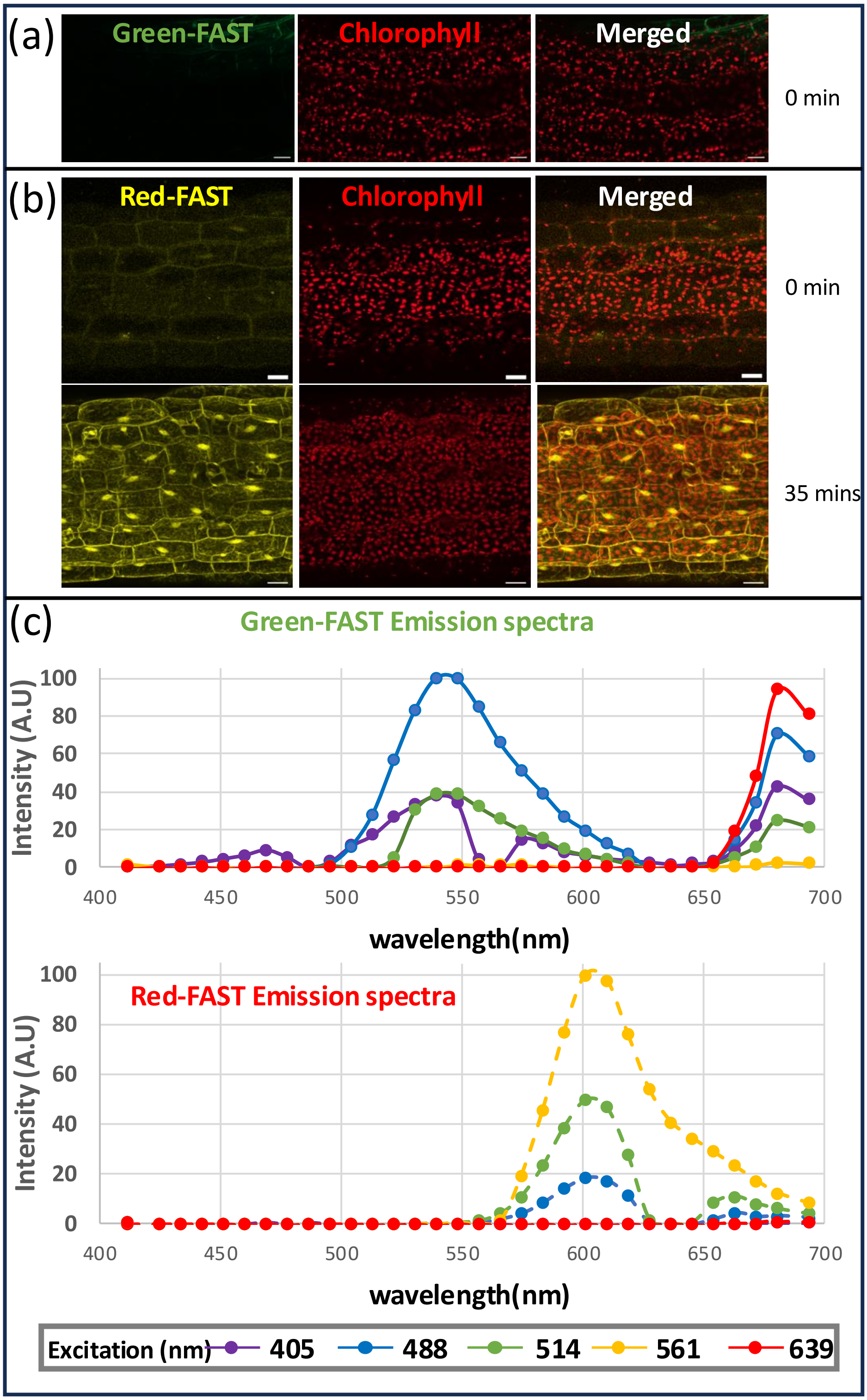
(a)
Green-FAST
Chlorophyll
Merged
0 min
(b)
Red-FAST
Chlorophyll
Merged
0 min
35 mins
(c)
Green-FAST Emission spectra
Intensity (A.U)
100
80
60
40
20
0
400
450
500
550
600
650
700
wavelength(nm)
Red-FAST Emission spectra
Intensity (A.U)
100
80
60
40
20
0
400
450
500
550
600
650
700
wavelength(nm)
Excitation (nm)
405
488
514
561
639

Figure S3

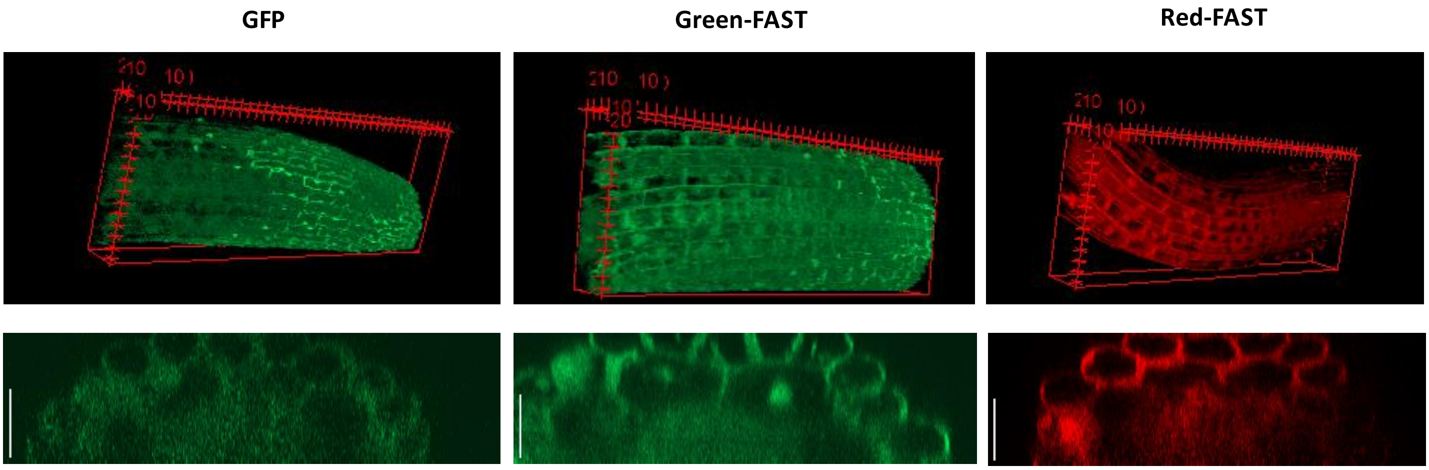

Figure S4

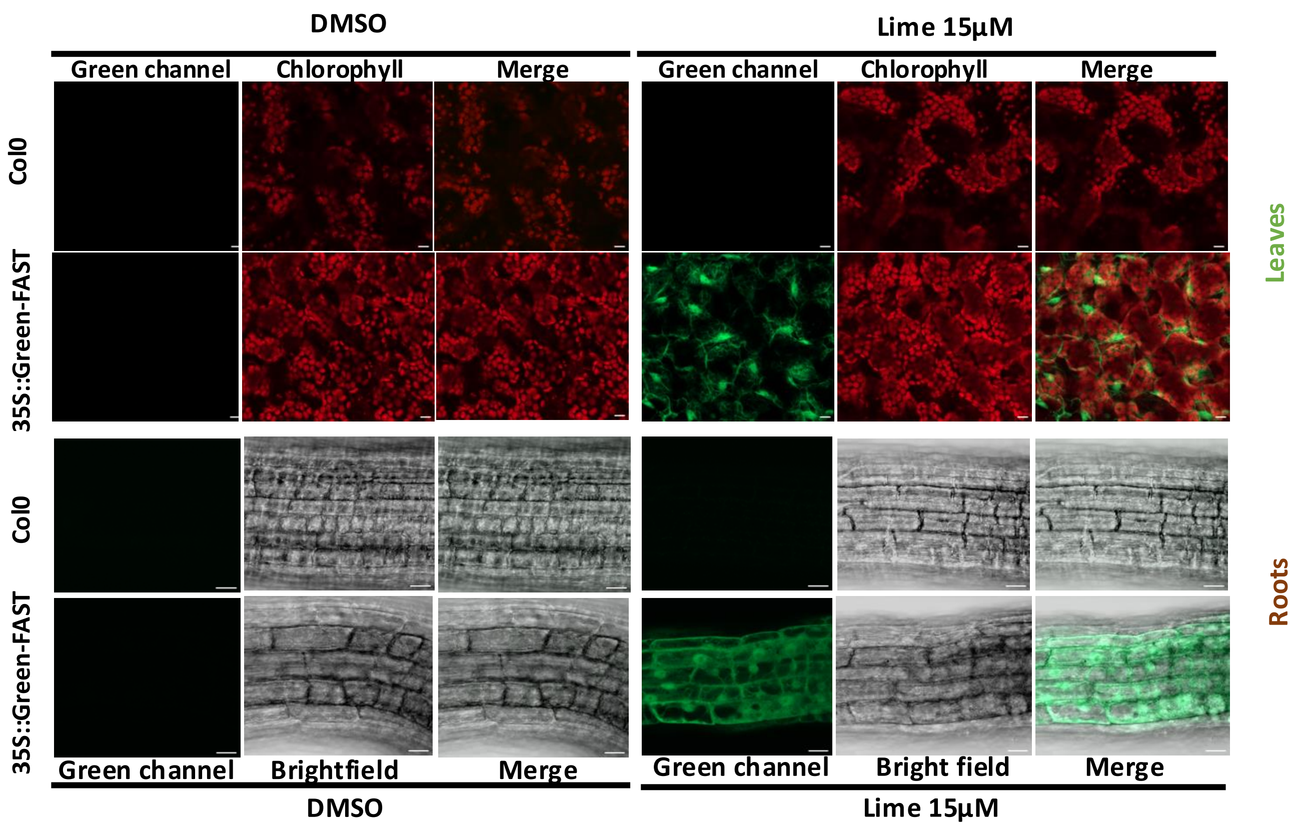
DMSO
Lime 15µM
Green channel
Chlorophyll
Merge
Green channel
Chlorophyll
Merge
Col0
35S::Green-FAST
Col0
35S::Green-FAST
Leaves
Roots
Green channel
Brightfield
Merge
Green channel
Bright field
Merge
DMSO
Lime 15µM

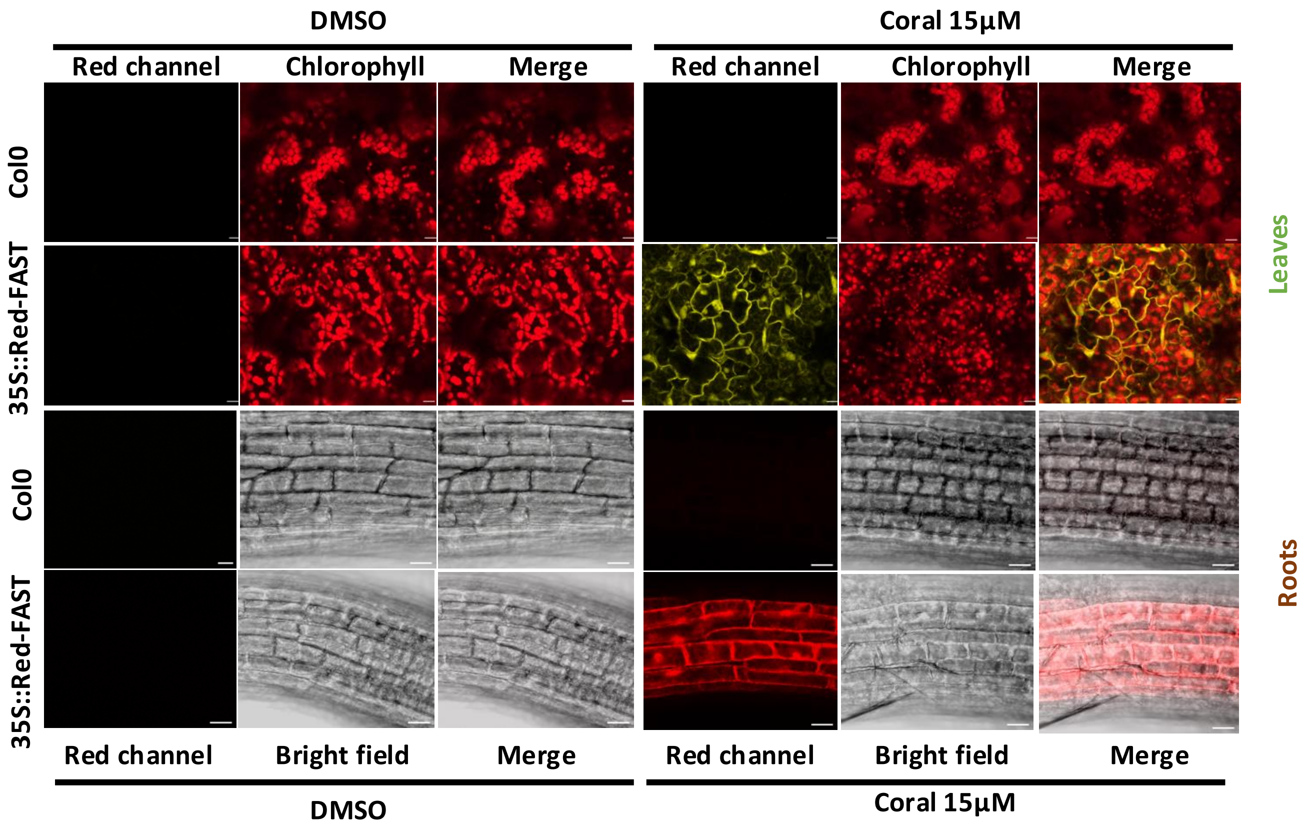
DMSO
Coral 15µM
Red channel
Chlorophyll
Merge
Red channel
Chlorophyll
Merge
Col0
35S::Red-FAST
Col0
35S::Red-FAST
Leaves
Roots
Red channel
Bright field
Merge
Red channel
Bright field
Merge
DMSO
Coral 15µM

Figure S5

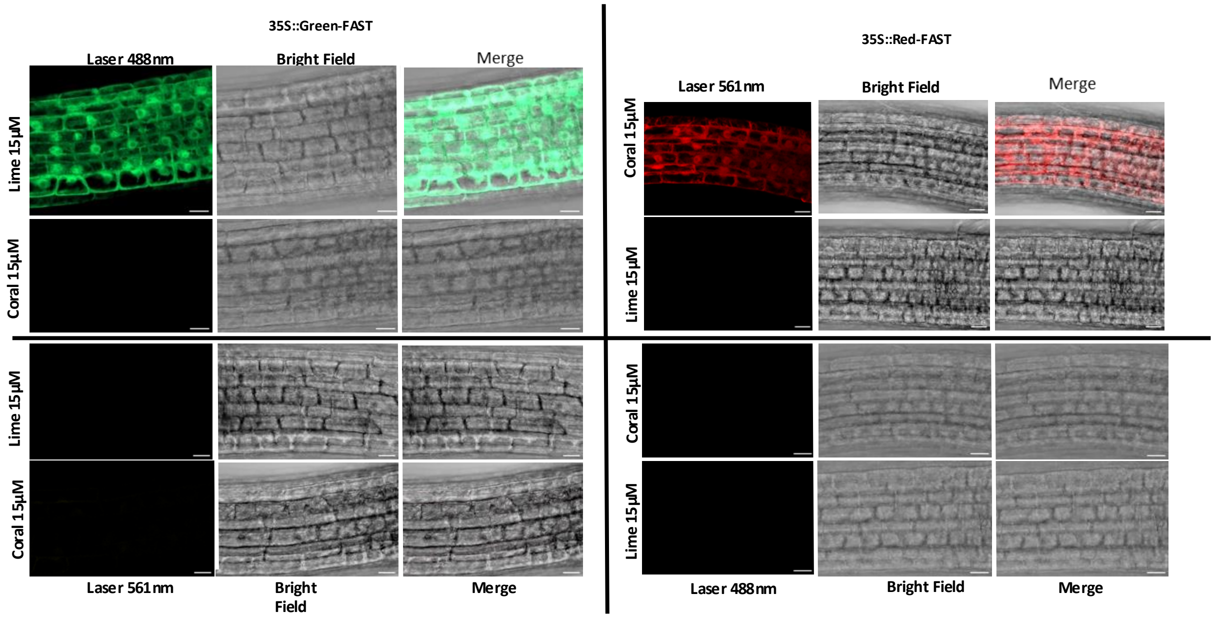
35S::Green-FAST
Laser 488nm
Bright Field
Merge
Lime 15µM
Coral 15µM
Lime 15µM
Coral 15µM
Laser 561nm
Bright Field
Merge
35S::Red-FAST
Laser 561nm
Bright Field
Merge
Coral 15µM
Lime 15µM
Coral 15µM
Lime 15µM
Laser 488nm
Bright Field
Merge

Figure S6

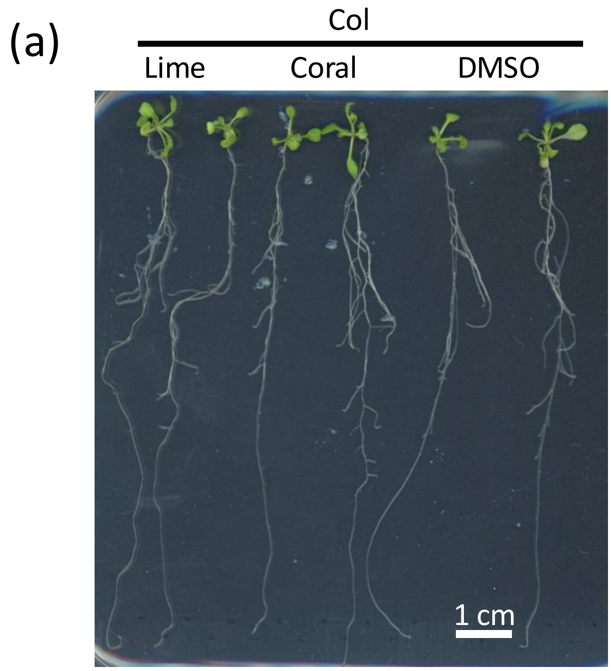

Genotype	Treatment	Primary root elongation per day (mm)	T-test
**Col**	DMSO	14.5 +/- 2.2	
**Col**	Coral	14.4 +/- 1.2	NS
**Col**	Lime	14.0 +/- 2.5	NS

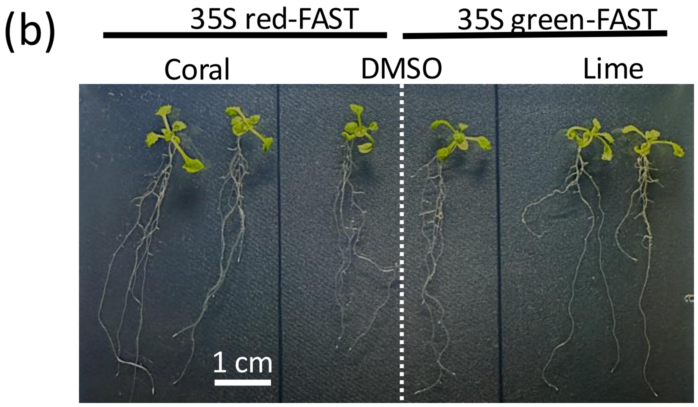

Genotype	Treatment	Primary root elongation per day (mm)	T-test
**Red-FAST**	DMSO	11.9 +/- 1.7	
**Red-FAST**	Coral	12.7 +/- 1.3	NS
**Green-FAST**	DMSO	12.4 +/- 2.6	
**Green-FAST**	Lime	11.3+/-1.9	NS

Figure S7

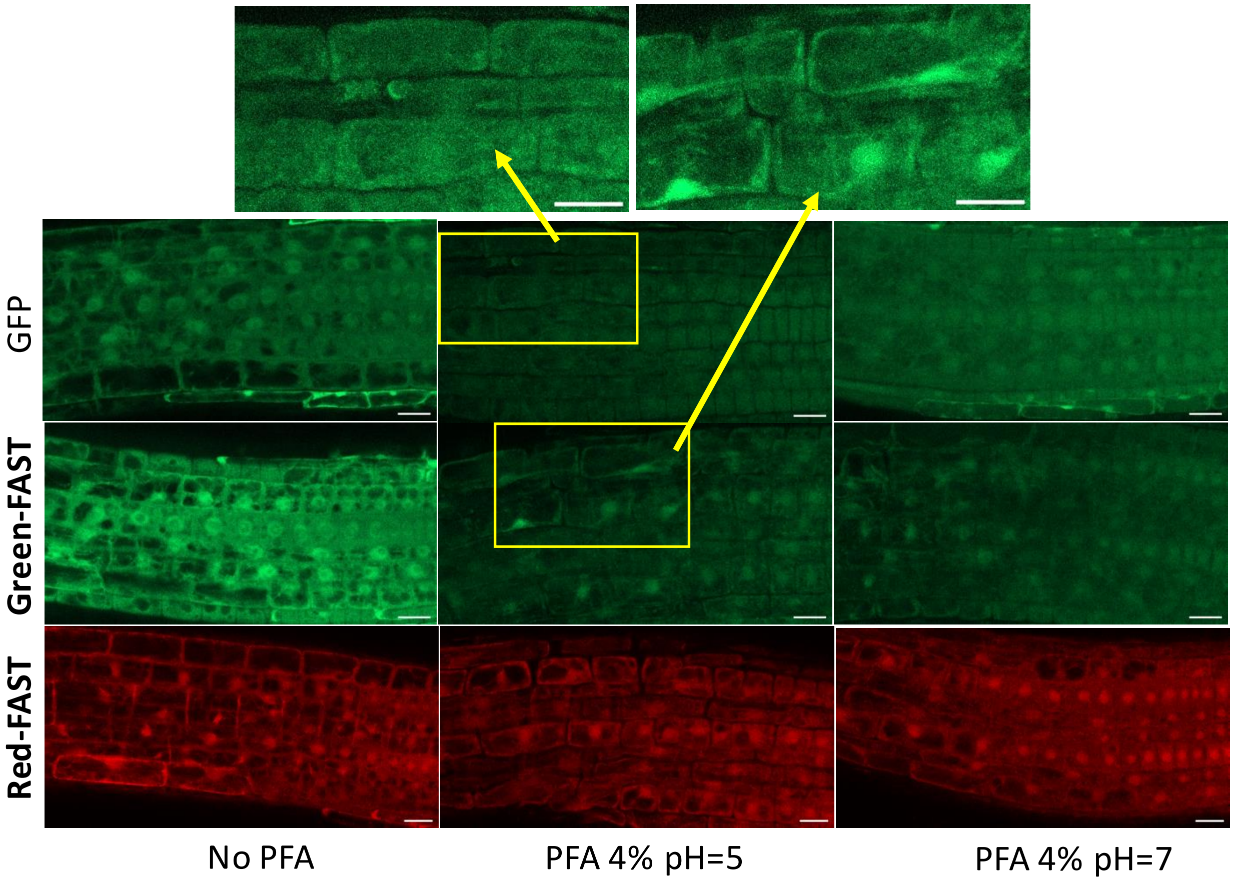

Figure S8

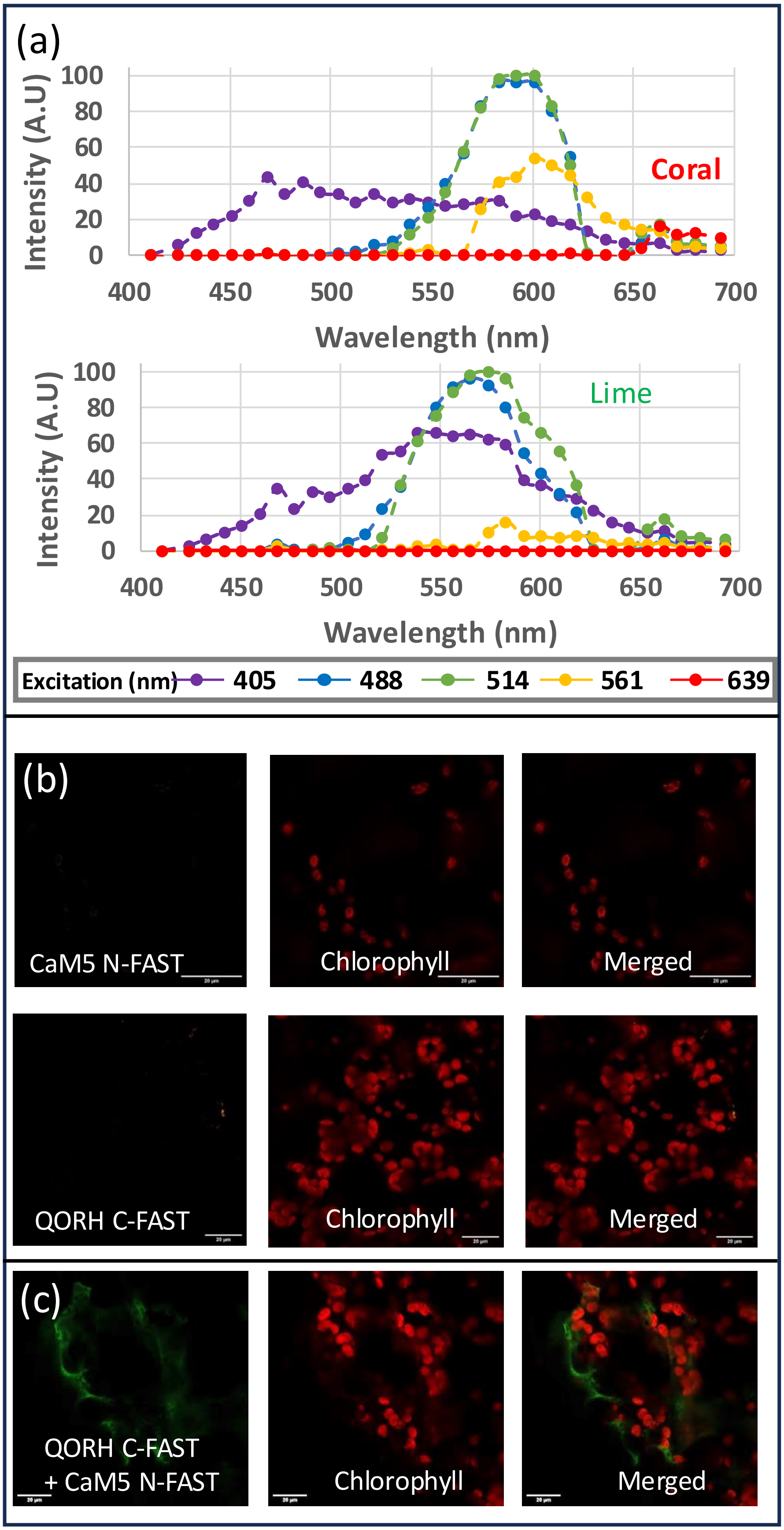
(a)
Intensity (A.U)
Coral
Wavelength (nm)
Lime
Excitation (nm)
405
488
514
561
639
(b)
CaM5 N-FAST
Chlorophyll
Merged
QORH C-FAST
Chlorophyll
Merged
(c)
QORH C-FAST
+ CaM5 N-FAST
Chlorophyll
Merged

Figure S9

ceQORH-C *RspA* Split FAST (Split FAST sequence underlined)

- GTCGACATGGCTGGAAAACTCATGCACGCTCTTCAGTACAACTCTTACGGTGGTGGCGCCGCCGGATTAGAGCATGTTCAAGTTCCGGTTCCAACACCAAAGA
GTAATGAGGTTTGCCTGAAATTAGAAGCTACTAGTCTAAACCCTGTTGATTGGAAAATTCAGAAAGGAATGATCCGCCCATTTCTGCCCCGCAAGTTCCCCTG
CATTCCAGCTACTGATGTTGCTGGAGAGGTCGTTGAGGTTGGATCAGGAGTAAAAAATTTTAAGGCTGGTGACAAAGTTGTAGCGGTTCTTAGCCATCTAGGT
GGAGGTGGACTTGCTGAGTTCGCTGTTGCAACCGAGAAGCTGACTGTCAAAAGACCTCAAGAAGTGGGAGCAGCTGAAGCAGCAGCTTTACCTGTGGCGGGTC
TAACCGCTCTCCAAGCTCTTACTAATCCTGCGGGGTTGAAGCTGGATGGTACAGGCAAGAAGGCGAACATCCTGGTCACAGCAGCATCTGGTGGGGTTGGTCA
CTATGCAGTCCAGCTGGCAAAACTTGCAAATGCTCACGTAACCGCTACATGTGGTGCCCGGAACATAGAGTTTGTCAAATCGTTGGGAGCGGATGAGGTTCTC
GACTACAAGACTCCCGAGGGAGCCGCCCTCAAGAGTCCGTCGGGTAAAAAATATGACGCTGTGGTCCATTGTGCAAACGGGATTCCATTTTCGGTATTCGAAC
CAAATTTGTCGGAAAACGGGAAGGTGATAGACATCACACCGGGGCCTAATGCAATGTGGACTTATGCGGTTAAGAAAATAACCATGTCAAAGAAGCAGTTAGT
GCCACTCTTGTTGATCCCAAAAGCTGAGAATTTGGAGTTTATGGTGAATCTAGTGAAAGAAGGGAAAGTGAAGACAGTGATTGACTCAAAGCATCCTTTGAGC
AAAGCGGAGGATGCTTGGGCCAAAAGTATCGATGGTCATGCTACTGGGAAGATCATTGTCGAGCCATCTGGAGGAGGAGGATCCGGAGGAGGAGGATCAATGG
GAGATTCTTTTTGGATTTTTGTTAAGAGACTTTAAGCGGCCGC

- **Mut2_ceQORH-C *RspA* Split FAST (Split FAST sequence underlined)**

- GTCGACATGGCTGGAAAACTCATGCACGCTCTTCAGTACAACTCTTACGGTGGTGGCGCCGCCGGATTAGAGCATGTTCAAGTTCCGGTTCCAACACCAAAGA
GTAATGAGGTTTGCCTGAAATTAGAAGCTACTAGTCTAAACCCTGTTGATTGGAAAATTCAGAAAGGAATGATCCGCCCATTTCTGCCCCGCAAGTTCCCCTG
CATTCCAGCTACTGATGTTGCTGGAGAGGTCGTTGAGGTTGGATCAGGAGTAAAAAATTTTAAGGCTGGTGACAAAGTTGTAGCGGTTCTTAGCCATCTAGGT
GGAGGTGGACTTGCTGAGTTCGCTGTTGCAACCGAGAAGCTGACTGTCAAAAGACCTCAAGAAGTGGGAGCAGCTGAAGCAGCAGCTTTACCTGTGGCGGGTC
TAACCGCTCTCCAAGCTCTTACTAATCCTGCGGGGTTGAAGCTGGATGGTACAGGCAAGAAGGCGAACATCCTGGTCACAGCAGCATCTGGTGGGGTTGGTCA
CTATGCAGTCCAGCTGGCAAAACTTGCAAATGCTCACGTAACCGCTACATGTGGTGCCCGGAACATAGAGTTTGTCAAATCGTTGGGAGCGGATGAGGTTCTC
GACTACAAGACTCCCGAGGGAGCCGCCCTCAAGAGTCCGTCGGGTAAAAAATATGACGCTGTGGTCCATTGTGCAAACGGGATTCCATTTTCGGTATTCGAAC
CAAATTTGTCGGAAAACGGGAAGGTGATAGACATCACACCGGGGCCTAATGCAATGTGGACTTATGCGGTTAAGAAAATAACCGGATCCGCAGCTCAGTTAGT
GCCACTCTTGTTGATCCCAAAAGCTGAGAATTTGGAGTTTATGGTGAATCTAGTGAAAGAAGGGAAAGTGAAGACAGTGATTGACTCAAAGCATCCTTTGAGC
AAAGCGGAGGATGCTTGGGCCAAAAGTATCGATGGTCATGCTACTGGGAAGATCATTGTCGAGCCATCTGGAGGAGGAGGATCCGGAGGAGGAGGATCAATGG
GAGATTCTTTTTGGATTTTTGTTAAGAGACTTTAAGCGGCCGC

- **CaM5-N *RspA* Split FAST (Split FAST sequence underlined)**

- GTCGACATGGCAGATCAGCTCACCGATGATCAGATCTCTGAGTTCAAGGAAGCCTTTAGCCTTTTCGACAAAGACGGAGATGGTTGCATCACAACGAAAGAGC
TAGGAACAGTGATGAGATCATTAGGTCAAAATCCAACAGAAGCAGAGTTACAAGATATGATAAACGAAGTAGATGCTGATGGTAACGGAACCATAGACTTCCC
TGAGTTTCTGAACCTAATGGCTAGGAAGATGAAGGACACTGACTCTGAAGAAGAGCTGAAAGAAGCCTTCAGGGTTTTCGATAAGGACCAGAACGGTTTCATC
TCGGCAGCTGAGTTAAGACATGTAATGACAAATCTTGGTGAGAAGTTAACTGATGAAGAAGTTGATGAGATGATCAAAGAAGCTGATGTTGATGGAGATGGTC
AGATCAATTATGAAGAGTTTGTCAAAGTTATGATGGCAAAGAGAAGAGGGAAGAGAGTTATGGCAGCAAAGCGTAGTAGCAATTCTGCTGAATACAAAGAAAA
GAATGGTCGCCGGAAAAGTCACTGCCGTATTCTCTCCGGAGGAGGCGGCAGCGGCGGAGGGGGATCCATGGAGACCGTGAGATTCGGCGGCGACGACATCGAG
AACAGCCTGGCCAAGATGGACGACAAGGCCCTGGACAAGCTGGCCTTCGGCGCCATCCAGCTGGACGGCAACGGCAAGATCATCCACTACAACGCCGCCGAGG
GCACCATCACCGGCAGAGACCCCAAGACCGTGATCGGCAAGAACTTCTTCACCGACGTGGCCCCCGGCACCCAGAGCAAGGAGTTCCAGGGCAGATTCAAGGA
GGGCGTGCAGAAGGGCGACCTGAACACCATGTTCGAGTGGATGATCCCCACCAGCAGAGGCCCCACCAAGGTGAAGGTGCACATGAAGAAGGCCATGACC TAA
GCGGCCGC

- **PHT1;3**(CDS without stop codon and *BpiI* restriction site) introduced in level 0 pAGM1287

- GAAGACAAAATGGCCGATCAACAGCTAGGAGTGCTAAAGGCACTTGATGTTGCGAAAACGCAACTTTACCATTTCACGGCTATTGTCATTGCCGGTATGGGCT
TCTTTACGGACGCGTACGATCTCTTTTGTGTGTCCTTGGTGACCAAGCTTCTTGGCCGCCTCTACTACTTCAATCCAACGTCAGCAAAGCCTGGCTCACTTCC
CCCTCATGTTGCGGCTGCAGTCAACGGTGTGGCCCTTTGTGGAACCCTTGCCGGTCAACTCTTCTTCGGATGGCTTGGTGACAAACTCGGACGGAAAAAGGTG
TACGGTATCACTTTGATCATGATGATTCTCTGCTCAGTTGCTTCCGGTCTTTCCTTGGGCAATTCGGCCAAGGGTGTCATGACGACTCTTTGCTTCTTCAGGT
TTTGGCTCGGGTTTGGCATTGGAGGTGACTACCCTCTATCTGCCACCATCATGTCTGAATACGCTAACAAGAAAACTCGTGGGGCTTTCATCGCGGCAGTGTT
CGCCATGCAAGGTGTAGGTATCTTGGCGGGAGGTTTTGTGGCACTTGCAGTTTCTTCCATATTTGACAAAAAGTTCCCATCGCCGACGTATGAGCAAGACAGG
TTTCTATCAACGCCTCCTCAAGCTGATTACATTTGGCGAATCATCGTCATGTTTGGTGCTTTACCCGCAGCTTTGACTTACTATTGGCGTATGAAGATGCCTG
AAACAGCCCGTTACACCGCTTTAGTTGCCAAGAACATCAAACAAGCCACAGCAGACATGTCCAAGGTCTTACAAACAGATCTCGAGCTTGAGGAAAGGGTGGA
GGATGACGTCAAGGACCCCAAAAAAAACTATGGCTTGTTCTCCAAGGAATTCCTTAGACGCCATGGGCTTCATCTCCTTGGGACTACCTCCACTTGGTTTTTG
CTTGACATCGCCTTCTACAGCCAAAACTTGTTCCAAAAGGATATTTTCTCGGCCATTGGATGGATCCCAAAGGCAGCCACTATGAACGCCATCCATGAGGTTT
TCAAGATTGCTAGGGCTCAGACTCTCATTGCCCTCTGCAGTACAGTCCCAGGTTACTGGTTCACAGTAGCCTTTATTGATATCATTGGAAGGTTTGCGATCCA
ACTAATGGGATTTTTCATGATGACCGTTTTTATGTTTGCTATTGCCTTCCCATACAACCACTGGATTTTACCAGATAATCGTATCGGATTCGTGGTTATGTAC
TCACTCACATTTTTCTTCGCCAACTTTGGACCCAATGCAACTACTTTCATTGTCCCAGCTGAAATCTTTCCAGCAAGGCTAAGGTCTACGTGCCATGGAATAT
CAGCCGCAACTGGTAAGGCTGGAGCCATCGTTGGAGCCTTCGGGTTCCTATATGCTGCTCAACCACAGGATAAGACCAAGACAGACGCAGGATACCCACCGGG
CATCGGAGTCAAGAACTCATTGATCATGCTTGGTGTCATTAACTTTGTTGGTATGCTCTTCACCTTCCTCGTCCCTGAGCCCAAGGGCAAGTCCCTTGAAGAA
CTCTCCGGCGAGGCTGAGGTTGATAAAGGTTCGTTGTCTTC

- **Red-FAST and linker** introduced in level 0 pICH41258

- GAAGACAAAATGGAGCATGTTGCCTTTGGCAGTGAGGACATCGAGAACACTCTGGCCAAAATGGACGACGGACAACTGGATGGGTTGGCCTTAGGCGCAATT
CAGCTCGATGGTGACGGGAATATCCTGCAGTACAATGCTGCTCAGGGAGACATCACAGGCGCAGATCCCAAACAGGTGATTGGGAAGAACTTCTTCAAGGATG
TTGCACCTGGAACGGATTCTCCCGAGTTTTACGGCAAATTCAAGGTAGGCGTAGCGTCAGGGAATCTGAACACCATGTTCGAATGGATGATACCGACAAACAG
GGGACCAACCAAGGTCAAGGTGCACATGAAGAAAGCCCTTTCCGGCGACAGCTTCTGGATCTTCGTGAAGAGACTGTCCGGAGGAGGCGGCAGCGGCGGAGGG
GGAGGTCCGTCTTC

- **Green-FAST and linker** introduced in level 0 pICH41258

- GAAGACAAAATGGAGCATGTTGCCTTTGGCAGTGAGGACATCGAGAACACTCTGGCCAAAATGGACGACGAACAACTGGATGGGTTGGCCTTTGGCGCAATTC
AGCTCGATGGTGACGGGAATATCCTGCAGTACAATGCTGCTGAAGGAGACATCACAGGCAGAGATCCCAAACAGGTGATTGGGAAGAACTTCTTCAAGGATGT
TGCAACTGGAACGGATTCTCCCGAGTTTTACCGCAAATTCAAGGAAGGCGTAGCGTCAGGGAATCTGAACACCATGTTCGAATGGATGATACCGACAAGCAGG
GGACCAACCAAGGTCAAGGTGCACATGAAGAAAGCCCTTTCCGGTGACAGCTATTGGGTCTTTGTGAAACGGGTGTCCGGAGGAGGCGGCAGCGGCGGAGGGG
GAGGTCCGTCTTC

Figure S10

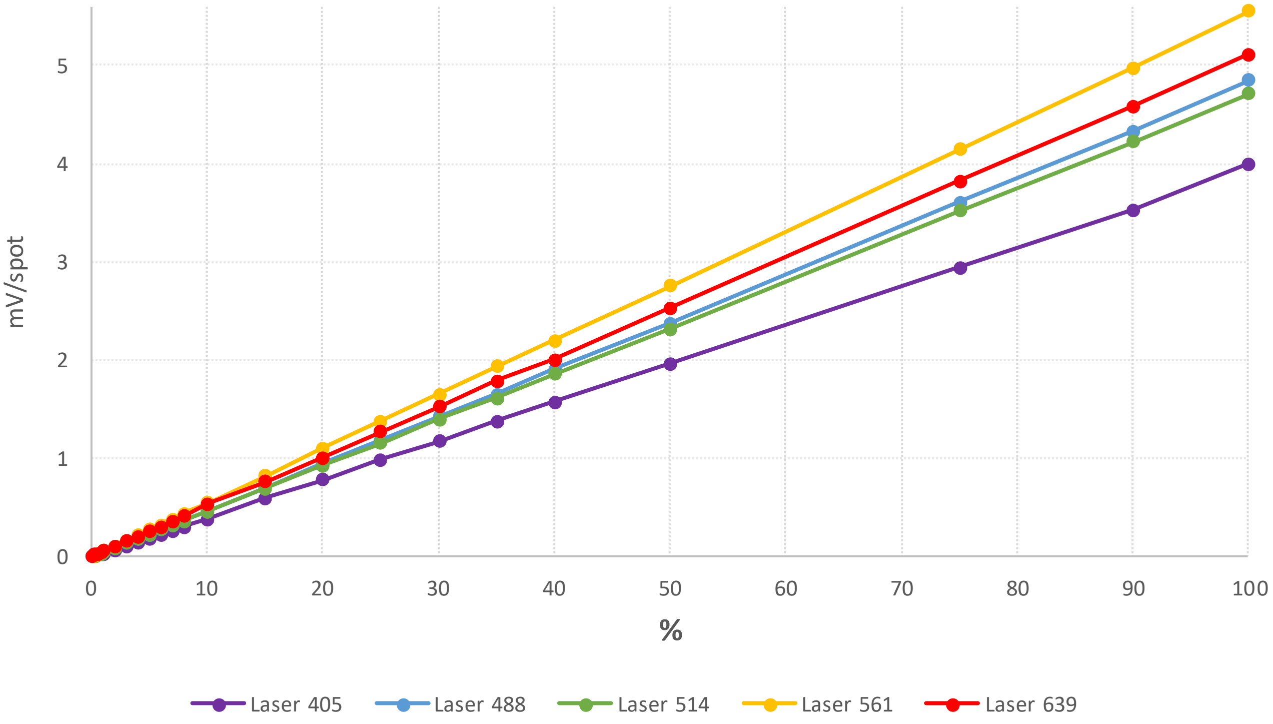

mV/spot
0
1
2
3
4
5
0
10
20
30
40
50
60
70
80
90
100
%
Laser 405
Laser 488
Laser 514
Laser 561
Laser 639
